# The α-Tubulin gene *TUBA1A* in Brain Development: A Key Ingredient in the Neuronal Isotype Blend

**DOI:** 10.3390/jdb5030008

**Published:** 2017-09-19

**Authors:** Jayne Aiken, Georgia Buscaglia, Emily A. Bates, Jeffrey K. Moore

**Affiliations:** 1 Department of Cell and Developmental Biology, University of Colorado School of Medicine, MS8108, 12801 E 17th Ave, Aurora, CO 80045, USA; JAYNE.AIKEN@ucdenver.edu; 2 Department of Pediatrics, University of Colorado Anschutz Medical Campus, Aurora, CO 80045, USA; GEORGIA.BUSCAGLIA@ucdenver.edu (G.B.); EMILY.BATES@ucdenver.edu (E.A.B.)

**Keywords:** TUBA1A, tubulinopathy, microtubule, neurodevelopment, lissencephaly, polymicrogyria

## Abstract

Microtubules are dynamic cytoskeletal polymers that mediate numerous, essential functions such as axon and dendrite growth and neuron migration throughout brain development. In recent years, sequencing has revealed dominant mutations that disrupt the tubulin protein building blocks of microtubules. These tubulin mutations lead to a spectrum of devastating brain malformations, complex neurological and physical phenotypes, and even fatality. The most common tubulin gene mutated is the α-tubulin gene *TUBA1A*, which is the most prevalent α-tubulin gene expressed in post-mitotic neurons. The normal role of TUBA1A during neuronal maturation, and how mutations alter its function to produce the phenotypes observed in patients, remains unclear. This review synthesizes current knowledge of TUBA1A function and expression during brain development, and the brain malformations caused by mutations in *TUBA1A*.

## 1. Introduction

Brain development is a highly complex process that requires careful coordination of cell proliferation, differentiation, migration, axon and dendrite growth and guidance, and synapse formation. Each of these cellular tasks rely on the proper regulation of microtubules, which are dynamic cytoskeletal polymers that help provide structure and generate force in all eukaryotic cell types. Neurons require elaborate networks of microtubules to support their complex morphologies and to perform a variety of critical functions. In developing neurons, dynamic microtubules are necessary to support essential functions such as morphological changes that underlie polarization and migration [1,2,3]. In particular, microtubules are key players in the initiation and extension of neurites, axon specification, and neuronal migration. In adult neurons, microtubules provide a stable backbone for established axons and dendrites, acting as essential trafficking routes for microtubule-based motors to carry various cargos from one compartment of the neuron to another. Microtubules also play an important role in synaptic plasticity, where they facilitate the morphological changes to dendritic spines that are thought to underlie synaptic enhancement and depression [4,5,6]. How microtubules are regulated to support distinct roles in different compartments of the neuron and at different stages of development to adulthood is widely unknown.

An emerging theme in the field is that microtubules may be regulated at the level of their protein building blocks. Microtubules are assembled from protein subunits known as tubulins, which are obligate heterodimers consisting of α- and β-tubulin polypeptides. Nearly all eukaryotes express multiple, different genes encoding α- and β-tubulin, known as isotypes. As many as nine α- and ten β-tubulin isotypes have been identified by genome analysis in humans [7,8]. One isotype, α-tubulin *TUBA1A*, is of particular importance to neural development [9,10,11], and will be the focus of this review. Throughout the history of tubulin research, the nomenclature describing *TUBA1A* has changed. Various names that have been used to describe the *TUBA1A* and *TUBA1B* isotypes are included in Table 1.

The strongest evidence for the critical role of tubulin isotypes in brain development comes from a growing number of heterozygous *de novo* missense mutations identified in isotypes of human patients with brain malformations, known as tubulinopathies [11,12,13,14,15,16,17,18,19,20]. The tubulin gene most commonly mutated in tubulinopathy patients is *TUBA1A*, the human isoform of α1 tubulin that is highly expressed in post-mitotic neurons [20,21]. Patients with mutations in *TUBA1A* present 5 classes of brain malformations including microlissencephaly, lissencephaly, and polymicrogyria, and a broad spectrum of clinical effects [20]. The spectrum of malformations suggests an important and complex role for TUBA1A in brain development, and mutations may alter TUBA1A protein function in ways that ultimately have drastically different impacts on brain development.

Exploring how TUBA1A contributes to neuronal function will provide important insight into how cells “tune” their microtubule networks using the programmed expression of a specific tubulin isotype. A major outstanding question in the microtubule field is how this tuning is achieved at the molecular level. Distinct tubulin isotypes could lead to outright functional differences due to intrinsic changes to the tubulin protein structure, which could alter microtubule dynamic instability or extrinsic binding of important microtubule-associated proteins (MAPs). Alternatively, carefully timed expression of a specific tubulin isotype could increase the concentration of tubulin present in the cell, supplying a necessary burst of new microtubule assembly during morphological changes such as axon outgrowth. In this review, we will discuss the current understanding of the regulation of microtubule function during neuronal development. We will particularly focus on *TUBA1A*, discussing its regulated expression, mutations associated with brain malformations, and how this α-tubulin might uniquely contribute to normal brain development.

## 2. Overview of Microtubules and Tubulin Isotypes

### 2.1. Microtubule Basics

**Figure 1:**
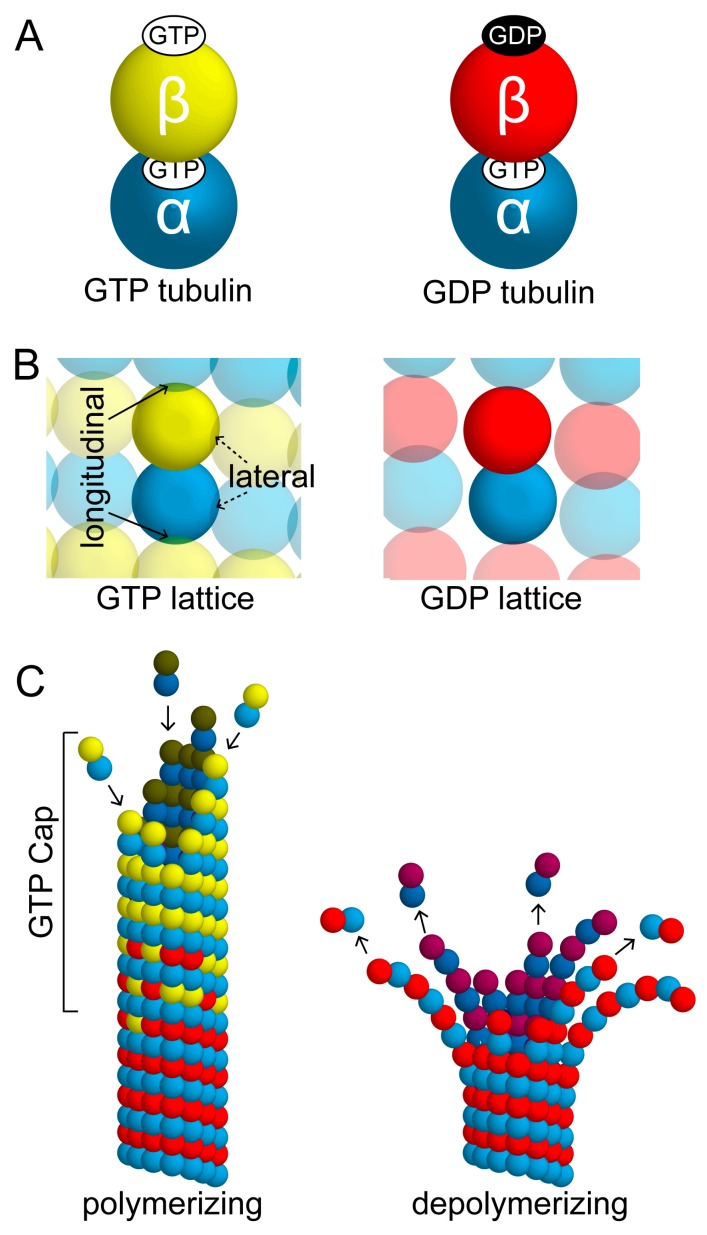
Microtubule structure and dynamics. (**A**) Heterodimer conformation in GTP and GDP states; (**B**) lattice conformation with labeled longitudinal and lateral interfaces; and (**C**) Microtubule conformation during polymerization and depolymerization.

Tubulin, the fundamental protein subunit of the microtubule, is a heterodimer of α- and β-tubulin polypeptides. α- and β-tubulins are conserved across eukaryotic evolution and are related to filament-forming proteins in prokaryotes and bacteriophage [22,23]. These proteins share the common characteristic of polymerizing into dynamic filaments, and this characteristic depends on their ability to bind and hydrolyze GTP. The tubulin heterodimer binds to two molecules of GTP at two separate sites. The so-called “non-exchangeable” or “N-site” is located at the intradimer interface, between α- and β-tubulin [24] (Figure 1A). GTP bound to the N-site is not hydrolyzed and exchanges at a slow rate [25]. In contrast, the “exchangeable”, or “E-site”, is located at the interdimer interface formed by the β-subunit of one heterodimer and the α-subunit of a neighboring heterodimer [24] (Figure 1A). GTP at the E-site can be hydrolyzed to GDP and subsequently exchanged for a new GTP nucleotide [26]. Importantly, the nucleotide status at the E-site plays a determining role in the conformation of the heterodimer and its interactions with other heterodimers (see description of “maturation” below) [27,28,29]. These changes alter tubulin activity by either affecting the conformation of the free heterodimer [30] or when it is packed into an microtubule [28,31].

α/β-Heterodimers polymerize into a sheet-like conformation, known as the microtubule lattice (Figure 1B). The lattice consists of longitudinal chains of heterodimers arranged head-to-tail, called protofilaments. Longitudinal interactions involve an extensive hydrophobic interface between α-tubulin of one heterodimer and β-tubulin of the adjacent heterodimer, which completes the E-site [32]. Protofilaments bind along their lateral sides to other protofilaments. Lateral interactions involve α-tubulin of one protofilament binding to the α-tubulins of neighboring protofilaments, and β-tubulin binding to neighboring β-tubulins. In contrast to longitudinal interactions, the interfaces of lateral interactions consist of flexible loop regions of α- and β-tubulin and feature prominent electrostatic contributions [28,32]. The binding energy of lateral interactions is predicted to be much weaker than longitudinal interactions, based on computational modeling [33,34]. In most cells, the lattice consists of 13 protofilaments that close into a cylindrical filament—the microtubule (Figure 1C). The closed cylindrical confirmation of the lattice restricts heterodimer addition or loss (i.e., polymerization or depolymerization) to the ends of the microtubule.

Microtubules are unique among cytoskeletal filaments in that they exhibit a behavior known as “dynamic instability”, which is defined as stochastic switching between states polymerization, where heterodimers are added to the growing microtubule lattice, and depolymerization, where heterodimers leave the shrinking microtubule lattice. Dynamic instability is an intrinsic property of microtubules, and is based on changes in the interactions between heterodimers in the lattice. Current models suggest a mechanism involving the “maturation” of tubulin heterodimers in the lattice, which can be depicted in a step-wise manner: (1) an “immature” heterodimer with GTP bound to β-tubulin at the nascent E-site binds to the microtubule end; (2) the next heterodimer arrives at the microtubule end and binds to the exposed β-tubulin of the first heterodimer, completing the E-site and stimulating GTP hydrolysis [26]; and (3) GTP hydrolysis triggers a wave of conformational changes (i.e., “maturation”) that compact the heterodimer and allosterically alter interactions with neighboring heterodimers [27,28,30,31]. At this point, the “mature” GDP-bound heterodimer has a weak affinity for the microtubule lattice and favors disassembly; however, the continued addition of new GTP-bound heterodimers at the growing end buries mature heterodimers within the lattice and prevents their escape. This layer of new GTP-bound heterodimers at the growing end is known as the “GTP cap” (Figure 1C). As long as the addition of new GTP-bound heterodimers outpaces the maturation of heterodimers in the lattice, the microtubule will continue to polymerize.

The switch from polymerization to depolymerization is known as catastrophe. Catastrophe is thought to be triggered when the GTP cap is exhausted; that is, when heterodimer addition slows and the cap matures to contain a threshold number of GDP-bound heterodimers. The structural changes in the microtubule that accompany catastrophe are poorly defined. However, cryoelectron microscopy of depolymerizing microtubules shows protofilaments forming “ram’s horns” that curl outward from the lattice [30,35] (Figure 1C). The curling of protofilaments away from each other suggests that conformational changes driven by maturation break lateral interactions between protofilaments before breaking longitudinal interactions within protofilaments. Lateral interactions are therefore likely to play an important role in the catastrophe mechanism, and are a key control point for regulating the organization and function of microtubule networks in cells.

Eukaryotic cells harness the dynamic instability of microtubules to construct highly organized networks for transporting cargoes and generating forces across large intracellular distances. The organization of microtubule networks and the generation of movement and force along microtubules is controlled by a diverse array of microtubule-associated proteins (MAPs). For comprehensive discussions on MAPs involved in different neuronal maturation stages, we refer readers to the following reviews [36,37,38,39]. Of particular interest for this review are the kinesins and dyneins—motor proteins that drive directional movement along microtubules.

Kinesins are a diverse family of ATPases that use energy from ATP hydrolysis to power directional movement along microtubules. The mechanism of kinesin motility involves the coordination of the head domains, which contain the ATP- and microtubule-binding activities, with adjacent neck linker domains that swing each head forward in an alternating, step-wise manner [40]. Among the 45 kinesin genes identified in mammals, several play important roles during brain development [41,42,43]. Dyneins represent a structurally and mechanistically different type of microtubule motor. Dynein also uses its ATPase activity to drive directional movement; however, the mechanism is very different from that of kinesin. The dynein mechanism involves a coordinated progression of conformational changes to mechanically link the nucleotide cycle of its AAA + ATPase domains to a microtubule-binding domain that is located ~15 nm away [44]. Furthermore, dyneins predominantly move toward the minus ends of microtubules, while most kinesins move toward the plus ends. Dynein motility is regulated by an assortment of dynein-binding proteins that regulate its speed, level of force production, and cargoes [45,46,47,48,49,50,51,52,53,54]. This regulation may explain how a single cytoplasmic dynein gene could be necessary for diverse roles in cells, while kinesins have undergone evolutionary diversification giving rise to 45 genes with specified functions. Because of the differences between dyneins and kinesins, it is likely that these motors use different mechanisms to bind and move along the microtubule surface. However, the contribution of the microtubule surface to motor activity and regulation is poorly understood.

Although much research in the microtubule field focuses on MAPs and motors that regulate microtubule networks by binding and moving along them, it is becoming clear that tubulin heterodimers themselves are intrinsic key regulators. Tubulins exhibit molecular differences that can be genetically encoded through different α- and β-tubulin genes, or conferred by posttranslational modifications. These molecular differences provide cells with a toolkit for changing the properties of microtubule networks.

### 2.2. Tubulin Isotypes

Nearly all eukaryotes express multiple, distinct genes for α- and β-tubulin, known as tubulin isotypes. Recent analysis of the human genome identified nine α-tubulin isotypes and ten β-tubulin isotypes, along with dozens of pseudogenes [7,8]. In addition to their different chromosomal locations, isotypes can be distinguished by three features: (1) the amino acid sequences they encode; (2) nucleotide sequences of the 3′ untranslated region (UTR); and (3) expression levels in different tissues or developmental stages. We will focus on key differences amongst the α-tubulin isotypes.

The amino acid sequence of α-tubulin is strongly conserved across eukaryotic evolution. For example, the human α-tubulin TUBA1A exhibits 86% sequence identity to α-tubulin in the unicellular eukaryote *Giardia lambia* and 75% identity to the α-tubulin in the budding yeast, *Saccharomyces cerevisiae*. Within the human α-tubulin isotypes (Table 2), there are a small number of amino acid sequence differences. Importantly, these differences are conserved in isotype homologues across species, suggesting that selective pressure may maintain isotype-specific sequence differences (Table 2). The majority of these differences are found in the 15–27 amino acids at the very carboxy-terminus; a region known as the Carboxy-Terminal Tail (CTT). CTTs decorate the outer surface of the microtubule, contain an abundance of amino acids with negatively-charged side chains, and are major sites of post-translational modifications (PTMs), which will be discussed in the next section. Current models propose that the molecular diversity generated by genetically-encoded differences and PTMs in the CTTs act as a “tubulin code” that regulates the activities of MAPs and motors at the microtubule surface [55,56]. Consistent with this model, several studies show that altering the amino acid sequence within the CTT causes changes in microtubule function in vivo [57,58,59,60,61]. Besides the CTTs, human α-tubulin isotypes also exhibit amino acid differences at other positions. For example, the human TUBA1A and TUBA1B isotypes have identical CTTs, but have different amino acids at position 232 and 340. Position 232 is buried deep within α-tubulin, while position 340 lies on the microtubule surface, near the interdimer interface [62]. Whether these amino acid differences lead to functional differences between TUBA1A and TUBA1B has not been investigated. This exemplifies a general deficit in our understanding of α-tubulin isotypes—although we have known of amino acid differences between α-tubulin isotypes for over 30 years, the functional consequences of these differences remain largely unexplored.

In contrast to the highly similar coding sequences of α-tubulin isotypes, the 3′-UTR regions are highly divergent. Vertebrate tubulin isotypes were originally identified from cDNA clones, and it was noted at that time that each isotype contained a distinct 3′UTR sequence [10,64,65]. What makes the differences in the 3′-UTR region especially intriguing is that they are conserved across species, with the 3′-UTR regions of human *TUBA1A* and *TUBA1B* sharing interspecies homology with rat *Tuba1a* and *Tuba1b*, respectively [64]. This conservation of the noncoding region implies selective pressure, and raises the question of how 3′-UTR regions contribute to function. Presumably, the 3′-UTR regions could differentially regulate mRNA stability and/or localization within a cell. A beautiful example of this regulation comes from studies in zebrafish, where the 3′-UTR of the β-tubulin isotype *tubb5* targets the mRNA to axons and distal growth cones during development [66]. This mRNA targeting could provide an appealing mechanism for increasing the supply of tubulin heterodimers at a region of the cytoplasm that is far from the nucleus. Whether the 3′-UTRs of α-tubulin isotypes provide similar regulation awaits discovery.

The third distinguishing feature of tubulin isotypes is their pattern of expression across different tissues and developmental stages. The β-tubulin isotypes have been extensively mapped to different tissues, cell types, and, in some cases, sub-cellular localization [67,68,69,70,71]. We have a comparatively poor understanding of the distributions of α-tubulin isotypes, with the exception of *TUBA1A*. *TUBA1A* is strongly and specifically expressed in the developing nervous system, and provides over 95% of the α-tubulin in the embryonic brain [10,72]. We will extensively discuss *TUBA1A* expression and its regulation in subsequent sections. Spatial and temporal expression data on α-tubulin isotypes remain sparse and may need to be readdressed. Tracking the expression of tubulin isotypes at the protein level is particularly challenging due to the high degree of homology, and will benefit from new approaches to selectively label isotypes without impairing their functions.

Why different cell types express specific tubulin isotypes is a long-standing question. On one hand, the coding and non-coding differences between isotypes could impart functional differences within microtubule networks. The strongest evidence for specific functional roles for α-tubulin isotypes comes from studies of in *Drosophila* and *C. elegans*, which demonstrate isotype-specific requirements for generating proper axonemal structures within cilia and flagella [73,74,75]. These findings underscore the possibility that isotypes may play specific roles in building complex microtubule architectures and raise the question of whether specific isotypes could be required to build other complex microtubule structures, such as those in neurons. An alternative, but not mutually exclusive explanation for differential isotype expression is that it provides a convenient mechanism to regulate the levels of tubulin protein in a cell. This is a particularly important challenge considering that cells must balance the levels of α- and β-tubulin to form heterodimers, and excess monomer, particularly β-tubulin, can be toxic [76,77,78]. Studies investigating tubulin isotypes have provided some answers to these questions, but our overall understanding of isotype biology is still in its infancy.

### 2.3. Tubulin Post-Translational Modifications

In addition to different isotypes, the tubulin subunits can be regulated by diverse PTMs. Various PTMs have been identified on neuronal microtubules, including detyrosination/tyrosination, polyglutamylation, acetylation, and polyamination. In some cases, these PTMs can further amplify genetically-encoded differences between isotypes, since the modified amino acid residues on α- or β-tubulin are only found in a subset of isotypes. In this section, we will briefly summarize the current evidence of major classes of PTMs and their functional impacts on neuronal microtubules. For comprehensive reviews of tubulin PTMs, the reader is referred to several recent reviews [55,79,80].

Detyrosination/tyrosination refers to the enzymatic removal of tyrosine from the CTT of α-tubulin (detyrosination), and subsequent re-ligation (tyrosination). This tyrosine is genetically encoded by six α-tubulin isotypes in humans, including *TUBA1A* (Table 2). Although the enzyme that catalyzes the detyrosination reaction in vivo has not been identified, it is well-established that tyrosination is catalyzed by Tubulin Tyrosine Ligase (TTL), which exclusively acts on free heterodimers to ligate tyrosine onto a detyrosinated α-tubulin [81,82]. Antibodies that selectively bind to either tyrosinated or detyrosinated α-tubulin show that these PTMs can be differentially enriched on specific microtubules within a network, or specific regions of an individual microtubule, and appear to correlate with the age of the microtubule lattice [83,84,85]. Accordingly, neurons exhibit an enrichment of tyrosinated α-tubulin at regions containing more newly-assembled microtubules (e.g., neurites and the distal ends of axons) while detyrosinated α-tubulin is primarily enriched in regions with older and highly stable microtubules (the axon shaft) [86,87,88]. Neurons lacking the TTL enzyme undergo aberrant neuronal development, indicating an important role for the cycle of detyrosination/tyrosination [89]. Currently, there is little to no evidence that tyrosination status influences the intrinsic stability of a microtubule. In contrast, tyrosination status does impact the binding of various proteins to the microtubule surface. Cytoskeleton-Associated Protein Glycine-rich (CAP-Gly) domains selectively bind to EEY/F motifs, such as those found in the CTT of α-tubulins, and this binding strongly depends on the aromatic side chain of the terminal Y/F residue [58,90,91,92]. Detyrosination therefore inhibits the microtubule-binding activity of CAP-Gly-domains. CAP-Gly domains are found in several microtubule-associated proteins, including CLIP170, the tubulin binding co-factors TBCB and TBCE, and the p150^glued^ subunit of dynactin. Recent studies show that the binding of p150^glued^ and CLIP170 to tyrosinated tubulin promotes the initiation of retrograde dynein-dependent transport in vitro and at the distal ends of axons [93,94]. Thus, detyrosination/tyrosination provides a system for local control of microtubule-MAP interactions and transport within a microtubule network.

Polyglutamylation is the most abundant tubulin PTM within the brain and involves the addition of variable-length chains of glutamate residues to genetically-encoded glutamates in the CTT regions of α- or β-tubulin. Polyglutamylation was originally found to extend from α-tubulin peptide sequences that are only present in the TUBA1A and TUBA1B isotypes, with the glutamate chains extending from the γ-carboxyl group of the genetically-encoded glutamate at position 445 [63]. This may represent the primary polyglutamylation site on α-tubulin; however, alternative glutamates in the α-tubulin CTT may also be targeted for modification. The enzymes that catalyze polyglutamylation belong to the Tubulin Tyrosine Ligase Like (TTLL) family [95]. To date, twelve mouse TTLLs have been identified based on sequence homology, although only six of these exhibit polyglutamylase activity in vitro [96]. Among these, TTLLs 5, 6, 11, and 13 selectively modify α-tubulin [95,96,97]. The removal of glutamate residues is catalyzed by Cytosolic Carboxypeptidase (CCP) enzymes, which can either shorten polyglutamate chains or remove the genetically encoded, penultimate glutamate from detyrosinated α-tubulin, creating a truncated species known as ∆2-tubulin [98]. Similar to detyrosination/tyrosination, polyglutamylation is thought to alter the interactions of MAPs and motors at the microtubule surface. The clearest example is the regulation of microtubule severing by spastin. Here, the length of glutamate chains provides a tunable signal for directing spastin’s severing activity [99,100]. Although the mechanistic role of polyglutamylation in neurons is still unclear, it appears to be developmentally regulated and correlate with degeneration. Both in vivo and in vitro experiments show that levels of polyglutamylation vary in different brain regions and increase over development, reaching their highest level in mature neurons [101]. Loss of function mutations affecting the CCP1 enzyme disrupt the normal program of tubulin PTMs in mouse neurons, increasing the amount of polyglutamylated tubulin and decreasing the ∆2-tubulin species [98]. CCP1 disruption causes Purkinje cell degeneration in mice, in a manner that depends on increased polyglutamylation [102,103,104]. This suggests an important yet poorly understand role for regulated polyglutamylation in neurons.

The presence or absence of specific PTMs can directly affect the stability of the microtubule. For example, the adult brain contains a much higher cold stable population of microtubules than the developing brain, and this increase in the proportion of cold stable microtubules in the brain has been attributed to an accumulation of polyamination on neuronal microtubules [105,106]. Polyamination describes the addition of polyamine to multiple, genetically-encoded glutamine residues in α- and β-tubulin, by the transglutaminase enzyme TG2 [106]. Polyamination is sufficient to stabilize microtubules in vitro, and TG2 protein and activity levels increase postnatally, suggesting a role in neuronal maturation [106]. Acetylation of α-tubulin also correlates with microtubule stability, and staining with antibodies to acetylated tubulin is commonly used as a measure of microtubule stability in cells. However, while long-lived microtubules tend to be acetylated, the direct impact of acetylation on microtubule stability is not well established. Two recent studies indicate that acetylation of α-tubulin may promote microtubule stability through an unexpected mechanism—softening the microtubule lattice and allowing it to withstand bending without breaking and depolymerizing [107,108]. Studies in *C. elegans* demonstrate that microtubule acetylation is necessary to form specialized microtubules with 15-protofilament lattices in touch receptor neurons [109,110]. In addition to direct effects on the microtubule lattice, acetylation has been reported to promote the activities of kinesin-1 motors in vivo [111] and dynein motors in vitro [112]. This is intriguing, since the canonical acetylation site at lysine residue 40 of α-tubulin is located on the luminal side of the microtubule cylinder. How acetylation impacts lattice stability, interactions on the microtubule surface, and the larger implications of microtubule acetylation in vivo remain active areas of research.

## 3. Roles of Microtubules during Neuronal Development and Adulthood

**Figure 2:**
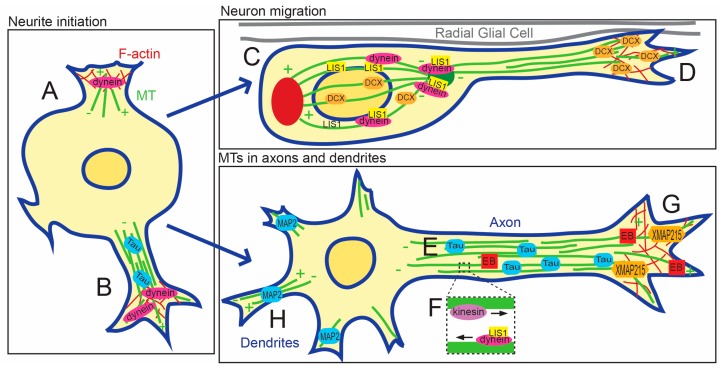
Overview of microtubule tasks during neuron maturation: (**A**) During neurite initiation, microtubules invade nascent lamellipodium; (**B**) microtubules form bundles to stabilize neurites; (**C**) microtubules form a perinuclear cage and provide force for nucleokinesis during neuronal migration; (**D**) in the migrating growth cone, microtubules stabilize and aid leading process growth; (**E**) polarized, bundled microtubules provide structural backbone of axon; (**F**) microtubules act as a transportation track for microtubule motors; (**G**) microtubules support axonal growth cone dynamics; and (**H**) microtubules of mixed polarity provide support to dendrites.

Microtubules play numerous important roles during brain development, particularly in neurons, where the microtubule cytoskeleton has been intensely studied. Once neuronal progenitors exit the cell cycle to become neurons, diverse microtubule-based maturation stages must occur for the neuron to correctly extend dynamic neurites, migrate to the proper position, form and guide long axons, set-up synapses, and sustain the diverse regions of the neuron. Microtubules are essential for these phases of neuronal maturation and are differentially regulated by numerous MAPs to perform diverse neuronal microtubule-based functions (Figure 2).

As shown in Figure 2, microtubules interact with numerous MAPs during each stage of neuronal maturation to properly perform various cellular tasks. During neurite initiation, dynamic actin forms lamellipodia that become stabilized by invading microtubules (Figure 2A) [113]. Interestingly, the dynamic properties of microtubules at this stage may inform which neurite becomes the future axon. Locally stabilizing microtubules in a particular neurite with stabilizing drugs results in that neurite becoming the axon [114]. Later, as neurites mature, microtubules become stabilized by MAPs such as tau and MAP2 (Figure 2B) [39,115]. The presence of tau helps identify the neurite as the axon, while MAP2 identifies a dendritic fate. At this stage, motors such as kinesin-1 and dynein are important in sorting and pushing microtubules to the end of the neurite [116].

As development proceeds, neuronal microtubules begin to exhibit a distinct polarity and decoration by MAPs depending on which type of process they inhabit. Microtubules within the axon become uniformly oriented with their plus ends directed away from the soma (Figure 2E), while dendritic microtubules retain a mixed polarity (Figure 2H) [117,118]. Dynein has been implicated in establishing and maintaining this plus-end-out (away from the soma) orientation in axons [116]. Plus-end-out polarity is critical for axonal transport, discussed below. The quantity and diversity of MAPs in the neuronal environment, and the interplay between them makes understanding each MAPs’ role difficult. For example, XMAP215 directly regulates microtubule plus-end dynamics in vitro, but in the growth cone it regulates the linkage of translocating microtubules to the F-actin network, thereby constraining microtubule growth velocity (Figure 2G) [119]. Future studies are needed to determine how microtubules are regulated throughout each stage of neuronal maturation, with attention given to how microtubule dynamics are regulated by the plethora of MAPs present during each developmental stage.

One of the most important functions of microtubules in adult neurons is to facilitate the efficient trafficking of organelles, proteins, mRNAs, and other cargoes across long distances in the axon. To appreciate the importance of efficient transport, consider that the distal regions of an axon can be as far as one meter away from critical protein and mRNA synthesis occurring in the soma. The unique microtubule polarity of the axon that is established early in development organizes transport in the anterograde and retrograde directions, with the help of motor proteins such as kinesin and dynein that move towards the plus or minus ends, respectively (Figure 2F). Kinesins, particularly kinesin-1 and 3 family members, carry cargoes towards the axon terminal by walking towards the plus ends of axonal microtubules [120]. Conversely, dynein carries cargoes back toward the soma by walking towards the minus ends [121]. The combination of uniform microtubule orientation and processive motility by directional molecular motors allows neurons to effectively transport cargoes across large distances.

In addition to establishing the architecture and transport networks within individual neurons, microtubule function is critical for neuronal migration, which is required to form the layers of the cortex and many other structures in the brain. During neuronal migration, microtubules extend into the leading process where they help to steer the protrusive growth cone at the end of the axon (Figure 2D) [122]. Microtubules also generate force to move the nucleus, in a process known as nucleokinesis. Here, microtubules extend back from the centrosome to form a cage-like network around the nucleus [123]. Dynein and its regulator LIS1 generate pulling forces to draw the nucleus toward the centrosome and move the centrosome toward the leading process [124]. Consistent with the important roles of the microtubule network in neuronal migration, mutations in LIS1 and the microtubule regulator doublecortin/DCX are associated with migration disorders that give rise to brain malformations [125].

## 4. Tubulinopathies Reveal Essential Role of TUBA1A in Brain Formation and Function

Recent studies investigating the genetic cause of brain malformation disorders have revealed that heterozygous, missense mutations to *TUBA1A* and other neuronal tubulin isotypes play an important role in brain development. First shown by Keays et al. in 2007, sequencing of tubulin genes has proven fruitful in uncovering the genetic source of numerous patients exhibiting complex neurological and physical phenotypes with cortical malformations. From these genotype-phenotype analyses, *TUBA1A* is recognized as vital for neurodevelopment based on the devastating effects observed in patients containing heterozygous, *de novo TUBA1A* missense mutations [11,12,13,14,15,16,17,18,19,20]. Brain malformation disorders caused by mutations to *TUBA1A* and other neuronally expressed tubulin isotypes are collectively termed “tubulinopathies”, and lead to severe cortical abnormalities, mental retardation, and commonly epilepsy and paralysis [11,12,13,14,15,16,17,18,19,20]. Patients containing *TUBA1A* mutations exhibit a wide variety of cerebral cortex malformation phenotypes including lissencephaly, pachygyria, microlissencephaly, and polymicrogyria. While known genetic causes of these phenotypes hint at which developmental processes may be disrupted, little is known about how the *TUBA1A* mutations disrupt microtubule functions, or even how these disruptions could cause the larger-scale cellular and tissue problems seen in patients. The wide variety of brain malformations observed in patients leads to the prediction that different missense mutations in *TUBA1A* may disrupt different neuronal maturation phases. Disease-causing mutations to *TUBA1A* therefore provide a valuable opportunity to investigate the numerous neurodevelopmental stages that require TUBA1A, and how microtubules must be regulated for each stage to occur appropriately.

**Figure 3:**
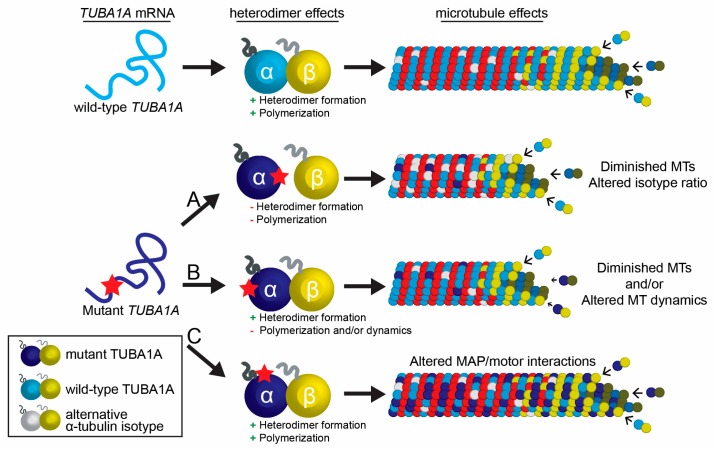
Potential consequences of TUBA1A mutations. TUBA1A mutations may lead to (**A**) protein folding defects or heterodimer instability; or (**B**) altered lattice interactions. Either of these defects may produce haploinsufficiency/loss of function consequences, resulting in fewer polymerization competent tubulin heterodimers available to form microtubules, or changes in microtubule dynamics. This also may lead to changes in the ratio of α-tubulin isotypes present in the microtubule lattice; (**C**) TUBA1A mutations may lead to mutant tubulin heterodimers that appropriately polymerize and cause toxic, gain of function consequences from within the microtubule lattice. Once in the lattice, mutant dimers may intrinsically change microtubule behavior or extrinsically alter MAP binding.

The identification of neurodevelopment disorder-causing *TUBA1A* mutations supports the hypothesis that this particular α-tubulin isotype is essential for neuronal maturation; however how can subtle changes to one α-tubulin protein lead to drastic changes in the formation of the brain? Expression studies of *TUBA1A* mRNA make it clear that *TUBA1A* is by far the most prevalent α-tubulin isotype in the embryonic nervous system, accounting for more than 95% of α-tubulin mRNA [72]. Thus, mutations to *TUBA1A* could greatly affect the available pool of neuronal tubulin. Tubulin mutations can act to alter numerous tubulin/microtubule characteristics and functions, but most of the possible changes fit into three general categories: (A) disrupting tubulin folding and heterodimer formation, thereby depleting the pool of α-tubulin that is competent to assemble microtubules; (B) disrupting polymerization activity and/or microtubule dynamics regulation, which could either deplete the pool of assembly-competent α-tubulin or alter dynamics once mutant heterodimers have formed the microtubule lattice; or (C) assembling appropriately into microtubules but altering microtubule function by disrupting interactions with MAPs and motors (Figure 3). Identifying which category *TUBA1A* mutations fit into will greatly increase our understanding of tubulinopathy disease progression. This task is not easy, however, as structure-function predictions for tubulin residues are rarely straightforward. Tubulin is a complex protein that interacts with numerous binding partners and undergoes long range conformational changes as part of its principle biochemical activity. Therefore, knowing the location of a change in the tubulin sequence does not necessarily give insight into its functional consequences. This underscores the need for functional studies that can test and refine structural predictions. However, despite the growing list of tubulinopathy mutations, few studies provide insight into how individual mutations impact the tubulin protein and its function in vivo. Future studies must be performed to fill in the missing molecular, mechanistic steps between the known *TUBA1A* mutations and the final brain phenotype.

While few studies have specifically addressed how mutations that disrupt TUBA1A cause cortical malformations, understanding the known mechanisms can provide clues into how attributes of TUBA1A contribute to normal brain development. However, there is much more work to be done to elucidate the mechanistic role of TUBA1A. Several different aspects of TUBA1A function could go awry to disrupt cellular processes and ultimately lead to abnormal brain development. For example, interference with neuronal migration can lead to lissencephaly, but not all the aspects of TUBA1A that are important for neuronal migration are known. For example, a mutation to *TUBA1A* could cause haploinsufficiency through many mechanisms, including disruptions to TUBA1A folding, heterodimer formation, or microtubule assembly. This could disrupt neuronal migration due to a pool of incompetent tubulin dimers forming less stable microtubules. Alternatively, specific *TUBA1A* mutants could appropriately assemble into microtubules but then cause dominant changes that “poison” the microtubule network. The dominant disruption could be achieved through changes to microtubule dynamics or disruption of binding of MAPs involved in migration. The following sections attempt to marry the current understanding of cortical malformation progression with how identified *TUBA1A* mutations could potentially act in the established pathway.

### 4.1. TUBA1A Mutations Linked to Lissencephaly

Lissencephaly describes a set of cortical malformations where at least part of the brain surface appears smooth, lacking the cortical folds that are a hallmark of a healthy human brain [126]. Severe lissencephaly manifests as a complete lack of cortical folds (agyria), while milder forms present as fewer broad folds (pachygyria) or as bands of heterotopic gray matter embedded below the white matter of the cortex (subcortical band heterotopia (SBH)). All of these manifestations of lissencephaly are attributed to neuronal migration errors in the developing cortex. In agyria and pachygyria, neurons fail to reach their appropriate positions leading to a disordered four-layered cortical structure lacking gyri or sulci folds, instead of the normal, six-layered cortex containing folds. In SBH, cortical neurons inappropriately migrate to an area deep to the cortex, forming band-like patterns of grey matter beneath the cortex [127].

In principle, any perturbation to cortical migration could lead to lissencephaly phenotypes, but most identified cases are attributed to mutations to *LIS1* (also known as *PAFAH1B1*), *doublecortin/DCX*, and more recently *TUBA1A* [125]. These genes are encompassed by the cortical migration pathway, and provide the molecular basis for the malformation. LIS1 and DCX play important roles in regulating microtubule-based tasks. LIS1 is an adaptor for the microtubule-motor dynein, acting as a “clutch” that allows dynein to remain attached to microtubules for longer periods of time [128]. This modulation of dynein activity is important during neuronal migration, when dynein is responsible for pulling the microtubule-caged nucleus [129]. While LIS1 modulates dynein behavior, DCX binds directly to microtubules to stabilize and promote polymerization [130]. During migration, DCX facilitates the formation/maintenance of the microtubule cage around the nucleus, as well as stabilizing microtubules in the leading process of the migrating neuron [131]. These proteins are vital to migration, as disruption leads to defective migration, and overexpression of either LIS1 or DCX is sufficient to increase migration rates [131].

As lissencephaly-associated proteins are known to modulate the microtubule network, proper microtubule function must be requisite for correct cortical migration to occur. In fact, lissencephaly is the most prominent malformation attributed to *TUBA1A* mutations, with over 90% of patients exhibiting some form of lissencephaly malformation. This suggests that the consequence of many *TUBA1A* mutants is to disrupt migration in some way. The molecular basis of this disruption, or whether many different changes to tubulin function could lead to migrational defects, remains largely unknown. Table 3 provides the known *TUBA1A* mutations that cause lissencephaly.

### 4.2. TUBA1A Mutations Linked to Polymicrogyria

Polymicrogyria describes the cortical malformation characterized by excessive gyration (i.e., multiple, small folds) of the cerebral cortex [143]. The classification of this malformation is complex as cases of polymicrogyria are heterogeneous, with variable pathological results, clinical features, and etiologies. Even key characteristics used to define the malformation are controversial, as some sources point to abnormal cortical lamination as a key feature [144], while others affirm that cortical layering remains normal, and there are simply fewer neuronal populations inhabiting the layers [143]. A number of seemingly unrelated environmental and genetic causes have been implicated as the molecular basis of polymicrogyria, but the details of this developmental disorder remain mysterious.

The major non-genetic causes of polymicrogyria relate to ischemic insults in utero, such as hypoxia, hypoperfusion, congenital infections, and/or inflammation of the microvasculature [144]. Metabolic and mitochondrial diseases have also been implicated, with a mouse model of Zellweger syndrome revealing that polymicrogyria may be caused by defects in glutamate receptor-mediated calcium mobilization during neuronal migration [145]. Additionally, the transcription factor *PAX6,* which is important for neuronal migration and axon guidance, has been implicated in polymicrogyria. Contrary to these few hints implicating neuronal migration in polymicrogyria, the disorder is generally considered a “post-migrational” malformation, with issues occurring after neurons have completed their migratory pathway to form the cortical layers [143,146]. Polymicrogyria seems to be caused by numerous, seemingly unrelated etiologies that all result in an excessive gyration phenotype. This is supported by the identification of many genetic roots of the disorder, including signaling molecules, cytoskeletal elements, and others.

Contrary to lissencephaly, where cortical migration provides a clear culprit for the malformation, the complex etiology of polymicrogyria makes it difficult to predict how *TUBA1A* mutations contribute to the molecular basis of the disease. Polymicrogyria-causing *TUBA1A* mutations are significantly less common than lissencephaly-causing mutations (~13%), suggesting that perhaps only very specific disruptions of tubulin function can lead to polymicrogyria. Understanding how these TUBA1A mutations alter intrinsic properties of microtubules, interactions with MAPs, and consequently cellular functions will provide a window into the cellular progression of the disease, and will help shed light on the “post-migration” vs. “migration” debate. The *TUBA1A* mutations that lead to polymicrogyria are described in Table 4.

### 4.3. TUBA1A Mutations Linked to Microcephaly

Microcephaly describes a brain that is significantly smaller than average, typically measured by occipitofrontal circumference (OFC). Different definitions exist as to where the cutoff from small head to microcephaly occurs, with some defining it more stringently as less than 4 SD (i.e., below the 1st centile) below the average [148], while others include a broader range with less than 2 SD (i.e., below the 3rd centile) [149]. However, arguments have been made about the relevance of using such a broad label, as many infants within the −2 SD to −3 SD population will be “normal” [150]. Microcephaly can be categorized into two main divisions: primary microcephaly and secondary microcephaly. Primary microcephaly describes a non-progressive, significantly small head detected prior to 36 gestational weeks (GW), and generally results from reductions in neurogenesis or loss of neural stem cells [150]. The major causes of primary microcephaly include non-genetic, damaging events prior to birth or mutations to genes regulating mitosis in neuronal progenitors [148,150]. In contrast, secondary microcephaly describes when microcephaly progresses postnatally, and is considered a neurodegenerative condition [148]. Causes of secondary microcephaly are numerous and varied, encompassing anything that disrupts orderly development and function of the brain.

As *TUBA1A* is not expressed in neuronal progenitors, microcephaly associated with *TUBA1A* likely fits into the secondary microcephaly classification. Neuronal maturation relies on appropriate regulation of microtubules (Figure 2), so it follows that disrupting microtubule function could alter neuronal development and cause progressive microcephaly. This idea is supported by *TUBA1A* patient cases where head circumference measurements have been taken more than once, such as the patient with p.E27Q whose OFC decreased from within the normal range at birth (−1 SD) to microcephalic (−3.3 SD) by two months of age [134]. Table 5 provides *TUBA1A* mutations linked to microcephaly. In this review, we use the broadest definition of microcephaly and include cases where the OFC is 2 SD below the appropriate mean (i.e., less than the 3rd percentile). Using this classification, ~74% of *TUBA1A* mutations lead to “microcephaly”, or smaller than normal head size. It is important to note that microcephaly is never the primary cortical malformation associated with *TUBA1A* mutants, but is a common accompaniment to both lissencephaly and polymicrogyria cortical phenotypes.

### 4.4. TUBA1A Mutations Linked to Cerebellar Dysplasia

In addition to the cortical malformations described above, *TUBA1A* mutations also commonly cause dysplasia of a variety of other brain regions, most notably the cerebellum. In fact, “lissencephaly with cerebellar hypoplasia” is a common class of *TUBA1A* mutation-induced phenotype [12,16]. In addition, some identified cases hint that tubulin mutations may cause specific cerebellar phenotypes with only subtly disrupted, or normal, cortical folds [137]. Further sequencing of *TUBA1A* in patients without the characteristic cortical lissencephaly phenotype may prove fruitful in uncovering additional *TUBA1A* mutations.

### 4.5. Summary of TUBA1A Mutations Associated with Brain Malformations

In this section, we have merely provided broad categories of brain malformations linked to mutations in *TUBA1A*. Within each category are numerous mutations that lead to the identified cortical malformation, but also other detrimental neurological and physical phenotypes. The increasing number of *TUBA1A* mutations discovered and the growing list of phenotypic consequences call for a greater understanding of the mechanism(s) of tubulinopathy disease progression.

### 4.6. Cellular Impact of TUBA1A Mutations

While numerous studies have identified missense mutations in *TUBA1A* as the genetic basis of brain malformations, few studies have been conducted to determine how these mutations alter tubulin molecularly, or how the mutations alter neuronal maturation/function. Below we describe studies investigating the molecular and cellular impact of *TUBA1A* mutations.

Studies investigating the stability of the mutant tubulin heterodimer and its ability to incorporate into microtubules provide hints at the underlying mechanism of the mutant and point to whether they lead to haploinsufficiency (Figure 3A,B), or dominant disruption of microtubule function from within a polymerized microtubule (Figure 3C), or potentially both. To date, Tian et al. published the most comprehensive and informative study on the consequences of *TUBA1A* mutations on heterodimer formation and stability [151]. TUBA1A mutant proteins were expressed in vitro to perform transcription/translation analysis in rabbit reticulocyte to test tubulin heterodimer yield. They discovered that p.L397P, p.V303G, and p.R402C lead to significantly reduced amount of unstable tubulin heterodimer, and that p.I188L, p.I238V, p.P263T, p.L286F, p.R402H, and p.S419L caused slight reduction to the amount of tubulin heterodimer [151]. These data suggest that mutations such as p.P263T may act more dominantly, while p.L397P, p.V303G, and p.R402C may act more as haploinsufficient mutants. This hypothesis is supported by the discovery that when ectopically expressed, p.P263T can incorporate into microtubules, dampen microtubule dynamics in COS-7 cells, and decrease microtubule growth in neurites of E15.5 primary cortical neurons 1 DIV [16,151]. These data all point to p.P263T acting dominantly, as described by Figure 3C. Alternatively, when p.V303G was ectopically expressed, it caused no change in microtubule dynamics in COS-7 or primary cortical neurons [151], as might be expected from a mutant that leads to few competent tubulin heterodimers entering the microtubule (Figure 3A).

Few *TUBA1A* mutations that lead to lissencephaly have been satisfactorily linked to the migratory pathway. However, inferences can be made about the functions of TUBA1A that may be important for migration based on how MAPs interact with microtubules and on the limited molecular data available on some of the lissencephaly patient *TUBA1A* mutations. For instance, p.R264C mutation occurs frequently in the *TUBA1A* patient population (12.5%), causes the less severe pachygyria phenotype, and is one of the most investigated mutations. Molecular and cellular data interrogating the consequence of p.R264C reveal that the mutation reduces the frequency of heterodimer formation due to compromised folding efficiency [152]. This reduced folding efficiency does not completely cause tubulin heterodimers containing the p.R264C mutant to drop out of the polymerization-competent tubulin pool, as they have been seen to form microtubules in HeLa, COS-7, and P19 cells [16,151]. p.R264C has also been modeled using iPSCs derived from patient cells, revealing that neurospheres generated from the patient iPSCs are capable of normal neurite extension [138]. Taken together, these data reveal that the p.R264C *TUBA1A* mutation probably follows the mutant scenario outlined by Figure 3A, and more importantly that partial haploinsufficiency to *TUBA1A* is sufficient to disrupt cortical migration enough to cause pachygyria. Similarly, cellular studies with p.I188L, which causes laminar heterotopia, show that FLAG-tagged mutant Tuba1a incorporates into microtubules in P19 cells [16], but that the mutation also causes slightly reduced amounts of tubulin heterodimer formation [151].

Three other lissencephaly-causing mutations have been investigated for their effects on cellular function, p.N329S, p.C25F, and p.R64W. Mutation p.N329S causes lissencephaly with cerebellar hypoplasia [16,138], and when neurospheres were generated from patient iPSCs, the mutation caused abnormal neurite extension [138]. While these cellular data suggest that defective neurite extension leads to lissencephaly with cerebellar hypoplasia, how the microtubules are altered by the p.N329S mutation to cause this diminished neurite growth is unknown. Mutation p.C25F leads to lissencephaly/thin cortex, causes decreased microtubule density in mutant transfected COS7 cells, and also caused patient fibroblast microtubules to depolymerize faster in cold [133]. These results are hard to interpret without further molecular evidence, but suggest that p.C25F may cause a shift in the stability in microtubules and that this decrease in stable microtubules is also sufficient to derail cortical migration enough to cause lissencephaly. Mutation p.R64W leads to severe brain malformations, exhibiting extremely thin cerebral parenchyma, agenesis of the cerebellum, and additional devastating defects to other brain regions. Similar to p.C25F, this mutation also causes decreased microtubule density and unstable microtubules [133]. However, because the phenotypes of p.C25F and p.R64W are divergent, with R64W exhibiting much more severe brain malformations, additional data must be collected to distinguish the molecular consequences of these mutations.

Whereas several lissencephaly-causing mutations lead to a loss of microtubules in vivo, there are no examples of mutants acting dominantly to assemble into microtubules and “poison” their function. These gain-of-function effects could be exerted by mutations that alter amino acid residues on the microtubule surface, such as mutations of R402, which cause lissencephaly. No studies have yet investigated how the patient mutations p.R402C and p.R402H impact microtubule function in vivo; therefore, these are high priority for future investigation.

While a few mutations leading to lissencephaly have been examined in terms of molecular and cellular consequence, to our knowledge none of the polymicrogyria-causing *TUBA1A* mutants have been investigated. Understanding how these mutations alter microtubule function will be an important step in understanding the molecular basis of the polymicrogyria malformation.

In addition to the cellular data referenced above, two mouse models that contain *Tuba1a* point mutations have been developed. Mice with *N*-ethyl *N*-nitrosourea (ENU) induced mutations in *Tuba1a* have provide insights into how Tuba1a contributes to neuronal development. For example, an S140G substitution in Tuba1a interferes with efficient GTP binding and heterodimer formation, but heterodimers that include S140G mutant Tuba1a can incorporate into the lattice [11]. One copy of the S140G *Tuba1a* allele is sufficient to cause defective neuronal migration in the hippocampus and the auditory, visual, and somatosensory cortices [11]. This mutation increases neuronal branching and alters the direction of migration in the rostral migratory stream that populates the olfactory bulb [153]. The neuronal migration phenotypes caused by the S140G mutation can be rescued with increased expression of wild-type Tuba1a, indicating that this mutation reduces the function of the protein [11]. Intriguingly, in vitro data suggest that the S140G mutation cannot be rescued by expressing another α-tubulin isotype, Tuba8 [153]. Additionally, presence of the Tuba8 isotype significantly altered microtubule polymerization compared to control or S140G mutant cells. This study provides the first evidence of functionally distinct α-tubulin isotypes in neurons [153]. However, it is important to note that *Tuba8* expression has not been detected in neurons [154].

An N102D substitution in Tuba1a provides different insight into the role of Tuba1a in neural development. Homozygous *Tuba1a* N102D mice have significantly less acetylated tubulin in growth cones, suggesting that microtubules containing Tuba1a N102D are less stable in extending growth cones [155]. On a cellular level, homozygous *Tuba1a* N102D mice have defects in motor axon extension and synapse function [155]. On a tissue level, homozygous *Tuba1a* N102D mice have gross defects in brain development including disorganized cortical layers [155]. The asparagine residue at position 102 is highly conserved across eukaryotes, which allows for the impact of N102D to be isolated and tested in the simple model organism budding yeast. The N to D substitution in yeast α-tubulin interferes with heterodimer stability, impairs its ability to incorporate into the lattice, and increases the frequency of microtubule catastrophes/depolymerization events [155]. Interestingly, yeast cells expressing the mutant allele in one α-tubulin isotype appear to compensate by increasing the cellular levels of an alternative, wild-type α-tubulin isotype. This suggests a novel consequence of *TUBA1A* mutations—cells may respond by shifting the blend of α-tubulin isotypes, which could in turn alter the normal regulation of microtubule networks.

Together, these mouse models demonstrate that Tuba1a is important for axon extension, efficient synapse function, and migration of neurons during development. However, we do not understand how mutations that have been identified in tubulinopathy patients impact microtubule properties or cellular function to disrupt development. Genetic models of patient derived *Tuba1a* mutations would lend much needed insights into the attributes of Tuba1a that are important for its role in neurodevelopment.

## 5. TUBA1A Expression: A Burst of Tubulin to Fuel Morphogenesis?

To understand the critical role of *TUBA1A* in brain development, it is important to consider which cells express *TUBA1A*, and how expression is regulated.

### 5.1. TUBA1A Expression Pattern

*TUBA1A* was originally identified in a cDNA library from human fetal brain tissue [64]. Subsequent northern blot studies in cell lines showed that *TUBA1A* is expressed in neural-derived cells and is particularly abundant in cell lines with characteristic long cytoplasmic processes (such as adherent neuroblastomas), but not in cell lines that exhibit small, round morphologies [9]. Since then, numerous groups have tracked the expression of *TUBA1A* and its vertebrate homologues to neuronal populations after terminal mitosis, as they begin to extend long processes.

A series of classic studies elucidated the expression program of mouse *Tuba1a* mRNA across tissues and developmental stages. The earliest study investigating mouse *Tuba1a* expression via northern blot with probes recognizing the distinct *Tuba1a* 3′-UTR provides the most extensive inquiry into which organs express *Tuba1a* postnatally [10]. This study identified *Tuba1a* mRNA in the adult brain, lung, and at low levels in the testes; however, only alternative α-tubulin mRNAs were detected in the heart, kidney, liver, muscles, spleen, stomach, and thymus. A time course analysis of brain-derived samples revealed that *Tuba1a* is abundant through early postnatal stages, begins to decline by P10, but still is expressed beyond P32 through adulthood [10]. Subsequent studies sought to elucidate the program of *Tuba1a* expression in the brain; to our knowledge there are no other published studies confirming the presence of *Tuba1a* in the adult lung or testes.

**Figure 4:**
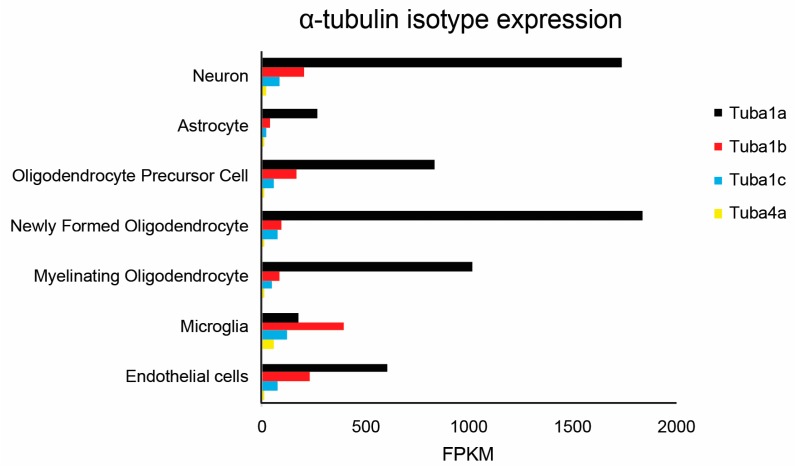
Dataset of RNA-Seq results for α-tubulin isotypes in mouse nervous system cell population. Distribution of α-tubulin isotype mRNA expression in various cell types generated from P7-17 mouse cerebral cortex. OPC population contains 5% microglial contamination. Adapted from [141].

Much of our understanding of *Tuba1a* expression in the developing brain comes from studies conducted by Miller and colleagues, who inserted lacZ behind the native *Tuba1a* promoter and used this *Tuba1a*:nlacZ transgenic mouse to monitor *Tuba1a* expression in vivo [156,157,158]. The expression profiles of mouse *Tuba1a* generated by these approaches, and later a transgenic reporter line using *Tuba1a* promoter-driven EYFP expression [159], are outlined in Table 6 and Table 7. These studies show that *Tuba1a* is expressed highly throughout the nervous system over the course of development, starting around E9.5 and continuing through early postnatal stages [10,156,158,159]. This trend holds true through the developing central and peripheral nervous system, with *Tuba1a* induction onset correlating to neurogenesis of numerous neuronal structures including the forebrain, midbrain, hindbrain, spinal cord, retina, and cranial nerves, among others [158]. Primary cultures generated from the transgenic reporter mice revealed that *Tuba1a* is expressed in cells positive for neuronal markers neuron-specific enolase (NSE) and βIII tubulin, but not in cells positive for stem cell or glial markers [156,158,159,160]. More recent RNA-seq studies from the Barres lab show that *Tuba1a* remains the most abundant α-tubulin isotype mRNA in neurons late in brain development (Postnatal Day 7) [154] (Figure 4). Based on these studies, the current model is that *Tuba1a* expression is induced coincident with or shortly after the terminal cell division that gives rise to neurons, and provides >95% of the α-tubulin mRNA in these cells [72].

While much of the RNA probing data suggest that *Tuba1a* expression is limited primarily to neurons, the recent RNA-seq dataset contends that *Tuba1a* may be expressed in more cell types than the earlier studies indicate [154]. As stated previously, numerous studies claim that *Tuba1a* mRNA is absent in stem cell, glial, and neuron progenitor populations, limited only to early born neurons [156,158,159,160]. However, the RNA-Seq data suggest that *Tuba1a* may in fact be expressed in numerous cell types found in the brain, with *Tuba1a* expression equally high in newly-formed oligodendrocytes as it is in neurons at Postnatal Day 7 (Figure 4). As immunohistochemistry double labeling studies suggest that oligodendrocytes in embryonic and adult mouse do not express *Tuba1a* [156,159,160], the contradictory expression of *Tuba1a* in glial populations calls for additional studies to conclusively uncover what cell types contain TUBA1A.

After brain development, *Tuba1a* mRNA levels persist, albeit at lower levels, in the adult mouse brain. Which adult cell populations express *Tuba1a* is somewhat controversial, due to conflicting data. Bamji and Miller found that *Tuba1a* mRNA was panneuronal in the adult mouse brain, with the highest levels found in neuronal populations that have the potential for morphologic growth, such as neurons of the piriform cortex [157] (Table 7). In contrast, Coksaygan and colleagues only detected *Tuba1a* mRNA in some regions of the adult brain (Table 7). How experimental differences might contribute to these discrepancies is not immediately clear.

While much of the *Tuba1a* expression data have been conducted in mouse, studies in rat models yield similar findings. Rat *Tuba1a* mRNA is highly enriched in the embryonic nervous system and is less abundant in adult brain [72]. Additionally, the rat data also support the idea that Tuba1a is induced in neurons undergoing neurite extension, such as in vivo at the cortical plate as well as in vitro in PC12 cells induced to differentiate with NGF treatment [72].

Interestingly, studies of the zebrafish *tuba1a* yield results that overlap with those from mammalian models, but with several important differences. Whereas zebrafish *tuba1a* is dramatically induced in the developing nervous system and declines coincident with the maturation, it is maintained at high levels in progenitor cells in the retinal periphery, lining the brain ventricles, and around the central canal of the spinal cord [161]. When cultured in vitro, these *tuba1a*-expressing cells divide and give rise to new neurons, suggesting that zebrafish may express *tuba1a* in neuronal progenitors as well as newly-born neurons [161]. Consistent with this notion, morpholino-based knock down of zebrafish *tuba1a* suppresses CNS development and leads to fewer differentiating neurons [162]. Intriguingly, Ramachandran et al. generated transgenic fish to conditionally and permanently label *tuba1a*-expressing cells, and found transient *tuba1a* expression in neural progenitors, skeletal muscle, heart, and intestine progenitors [163]. This experiment suggests that *tuba1a* expression is not neuronally limited in zebrafish.

These expression studies strongly suggest that *TUBA1A* is highly induced in neurons as they undergo cellular morphology changes to generate cytoplasmic processes. This suggests a simple model for why neurons, but not other cell types, use the conserved expression program for *TUBA1A—*neurons need significantly *more* α-tubulin at the time when they are maturing to form the long processes necessary to wire the nervous system.

### 5.2. Mechanisms Regulating TUBA1A Expression

While questions remain regarding which cell populations express *TUBA1A*, it is clear that expression is carefully regulated during development and adulthood. What pathways control this expression program for *TUBA1A*? A variety of factors and conditions that mediate *TUBA1A* mRNA production have been identified, and growth factors involved in neuronal differentiation play a prominent role.

Several studies have shown that growth factors are sufficient to stimulate *Tuba1a* expression. For example, PC12 cells from rats can be induced to differentiate into neurons with the addition of Nerve growth factor (NGF), and consequently *Tuba1a* mRNA levels increase as the differentiated PC12 cells begin to actively extend neurites [72]. Similarly, NGF treatment of neonatal rats causes a 5-10-fold increase of *Tuba1a* mRNA during a developmental period when *Tuba1a* levels normally decrease [164]. In addition to NGF, Fibroblast Growth Factor (FGF) and Platelet-Derived Growth Factor (PDGF) also stimulate *Tuba1a* expression. FGF was first shown to stimulate *Tuba1a* expression in rat sensory neurons, where treatment with FGF-1 increases *Tuba1a* mRNA levels by nearly ten-fold, and causes a concomitant increase in neurite outgrowth [165]. These findings suggest that exogenous stimuli such as growth factors may be sufficient to induce *TUBA1A* expression, at least in certain cell types.

**Figure 5:**
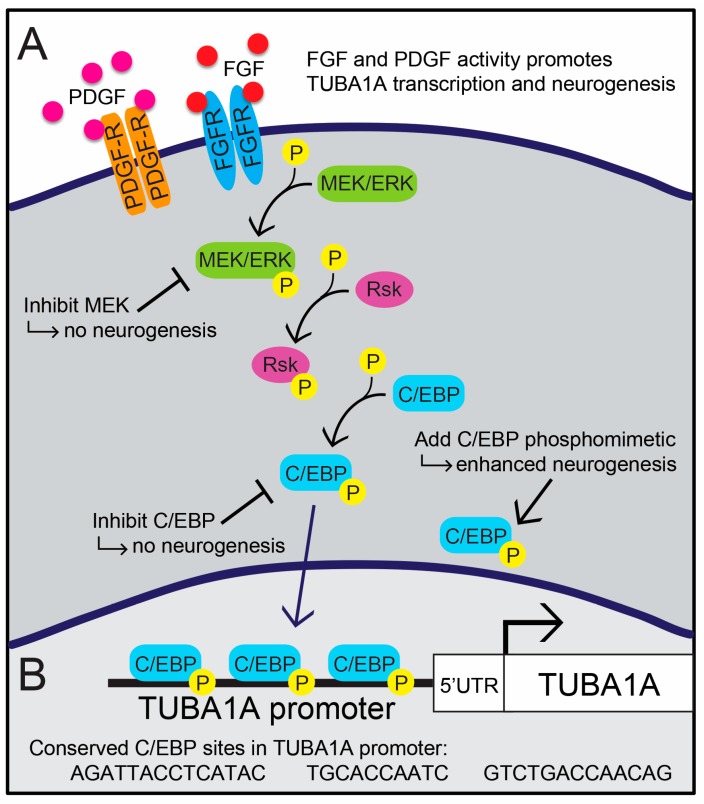
Transcriptional regulation of *TUBA1A* in mouse cortical progenitors. (**A**) FGF/PDGF treatment of cortical progenitors leads to a phosphorylation cascade activating MEK/ERK, Rsk, and C/EBP; and (**B**) in the nucleus, phosphorylated C/EBP directly binds three conserved C/EBP binding sites in the TUBA1A promoter, leading to transcription of TUBA1A and neurogenesis. Based on data from [153].

The most detailed evidence of the regulatory pathways that act downstream of growth factors to increase *Tuba1a* transcription comes from a study using mouse primary cultures of E12–E13 cortical progenitors [166]. This study revealed a phosphorylation cascade initialized by FGF and PDGF activation that ends in drastic upregulation of TUBA1A, and ultimately neurogenesis (Figure 5). Inhibiting factors in this pathway, MEK or C/EBP, leads to a significant decrease in *Tuba1a* expression and stops neurogenesis. Conversely, introducing an activated C/EBP phosphomimetic enhances *Tuba1a* expression and promotes neurogenesis. Furthermore, this study identified three C/EBP binding sites in the conserved TUBA1A promoter (Figure 5B), and revealed that direct C/EBP binding to these promoter regions greatly enhances TUBA1A transcription. Interestingly, inhibiting the *Tuba1a* neurogenesis pathway causes the would-be neurons to take on an astrocyte fate. This reveals that the same progenitors can be induced to become either neurons or astrocytes, and that *Tuba1a* activation is an important step in promoting the neuronal fate.

While the studies described above implicate common neurogenesis growth factors in *Tuba1a* expression, the specific requirements for *Tuba1a* activation remain largely unexplored. However, these data provide an additional link between the presence of Tuba1a and neurogenesis, further promoting the idea that Tuba1a may be an important regulator of neuronal fate.

### 5.3. TUBA1A in Regeneration

The unique temporal expression of *TUBA1A* during brain development indicates a critical, albeit poorly defined role for TUBA1A as a growth-associated neuronal α-tubulin. Interestingly, there is evidence that TUBA1A may also play important roles in remodeling the cytoskeleton during nerve regeneration and repair. Although *Tuba1a* mRNA expression declines after development, it is rapidly induced following nerve injury [72,167,168,169,170,171]. Mathew and Miller proposed that NGF could regulate *Tuba1a* expression following injury like in development [164,172]. The p75 NGF receptor is upregulated following axotomy, providing some support for this model [164,172]. The induction of a developmental α-tubulin, such as *TUBA1A*, during regeneration supports the idea that developing and regenerating neurons may utilize common mechanisms and that TUBA1A is of particular importance to growing neurons, regardless of age.

How might nerve injury trigger the re-emergence of *TUBA1A* expression? Several studies suggest that *Tuba1a* is normally repressed in adult neurons, but if normal processes are disrupted, *Tuba1a* expression is induced. For example, blocking fast axonal transport has been shown to induce *Tuba1a* expression [173,174]. These findings specifically implicate normal axonal transport as part of the mechanism that represses *TUBA1A*. For example, the neuron could be constantly monitoring its axonal integrity using intrinsic cues, such as backup of protein cargoes that would occur within the axon upon injury, or extrinsic cues such as constant uptake of growth factors like CNTF from nearby Schwann cells [167,173]. Alternatively, it has been proposed that repression could be maintained through physical contacts between neurons and neighboring cells [174]. However, current evidence does not support this model, as studies have found that loss of contact with either the basal lamina or the neuron’s target structure alone is insufficient to induce *Tuba1a* expression [167,172]. Although the exact mechanisms by which its expression is induced after nerve injury remain unclear, the model that *TUBA1A* expression may be controlled through mechanisms that monitor normal, homeostatic processes in neurons is intuitively appealing. This could explain how *TUBA1A* expression is repressed late in development, when these processes become active, and de-repressed when these processes are disrupted.

The expression of *TUBA1A* during regeneration after nerve injury provides an interesting opportunity to study regenerative capacity in neurons. While peripheral nerves are able to regenerate after injury, most neurons of the central nervous system do not have this capacity [175]. What allows peripheral axons to regenerate, while central axons cannot? Many of the initial studies identifying *Tuba1a* expression in response to injury focused on the peripheral nervous system; however, increases in *Tuba1a* expression have been noted following injury to central nerves as well [170]. This observation provides an opportunity to probe the differences in regenerative capacity between the central and peripheral nervous systems. Interestingly, one study found that *Tuba1a* gene expression after injury was broadly similar between peripheral nerves that regenerate and proximal central nerves that do not [170]. Importantly, in peripheral neurons, *Tuba1a* expression remained elevated until regeneration was complete. In central rubrospinal neurons, which did not regenerate, *Tuba1a* expression remained elevated for as long as two weeks post injury, even after levels of other injury-related cytoskeletal proteins had returned to normal [170]. However, if rubrospinal neurons are transplanted into a peripheral environment, they can regenerate [176]. Other studies have demonstrated that *Tuba1a* expression correlates with the timing of regeneration, and that in general *Tuba1a* will remain elevated until the axon is able to regenerate [172,177]. Thus, these data show that specific central neurons have regenerative capacity, but likely an unfavorable environment prevents complete regeneration. Conversely, study of a different population of central neurons found no evidence of *Tuba1a* upregulation after injury [169]. This may indicate some heterogeneity in regenerative capacity between different populations of central neurons; some neurons may lack the capacity to regenerate, regardless of whether the environment is conducive to regeneration [169]. Interestingly, *Tuba1a* can become upregulated after injury, even in uninjured regions of the nervous system. For example, elevated *Tuba1a* expression was not limited solely to injured peripheral axons, as neighboring uninjured axons can also display increased *Tuba1a* mRNA [172,177]. Additionally, elevated *Tuba1a* expression was found in the red nucleus, a brainstem nucleus that sends axons to the spinal cord, following a spinal cord compression injury [171]. The outlined studies show a selective upregulation of *Tuba1a* in regenerating neurons. The properties of TUBA1A α-tubulin that make it particularly suited to these functions are yet unknown. However, it is interesting to speculate that combinations of different tubulin isotypes could produce microtubules that are preferentially suited for specific functions.

## 6. Concluding Remarks

In recent years, the α-tubulin gene *TUBA1A* has emerged as a vital component in the development of the nervous system. With the identification of tubulinopathy-causing *TUBA1A* mutations, understanding how TUBA1A must function during neural development becomes an urgent inquiry. Microtubules perform many complex roles throughout development, but it remains unclear how TUBA1A, and the tubulin heterodimers and microtubules it produces, can be regulated to perform such diverse tasks in different neuronal compartments and through developmental and adult stages. The neuronal tubulin gene profile, as well as the poorly understood, yet remarkably complex, regulation of tubulin PTMs and MAPs may provide a starting point for determining how microtubules are tuned throughout neuron development. Brain malformation-causing *TUBA1A* mutations provide an irreplaceable opportunity to explore the role of microtubules at the cellular level of neuronal maturation, and at the tissue level of brain formation. While previous studies have performed the important task of sequencing tubulin genes to uncover underlying genetic mutations, future tubulinopathy studies must focus on the molecular and cellular consequences of these mutations to fully understand how altered TUBA1A function contributes to brain malformation progression. Discovering the molecular mechanisms behind TUBA1A mutations will be an important undertaking in cellular neuroscience, and could bring powerful new insight into the essential roles of microtubules in neural development.

## Figures and Tables

**Table 1:** α-tubulin protein isotype nomenclature.

	TUBA1A			TUBA1B		
Organism	Human	Mouse	Rat	Human	Mouse	Rat
Aliases	**TUBA1A** [7] **b-α-1** [7,12]	**Tuba1a** [7] **Tuba1** [7] **M-α-1** [10]	**Tuba1a** [7] **Tuba1** [7] **α-T14** [9] **T-α-1** [13]	**TUBA1B** [7] **k-α-1** [7,12]	**Tuba1b** [7] **M-α-2** [10] **Tuba2** [7]	**Tuba1b** [7] **T26** [13]

**Table 2:** Isotypes of α-tubulin.

Human Gene	Gene Accession	Protein Accession	Identity to TUBA1A	CTT Amino Acid Sequence	Mouse Gene	Identity to Human Isotype
*TUBA1A*	NM_006009	NP_006000	-	MAALEKDYEEVGVDSVEGEGEEEGEEY	*Tuba1a*	451/451
*TUBA1B*	NM_006082	NP_006073	449/451	MAALEKDYEEVGVDSVEGEGEEEGEEY	*Tuba1b*	451/451
*TUBA1C*	NM_032704	NP_116093	442/451	MAALEKDYEEVGADSADGEDEGEEY	*Tuba1c*	446/449
*TUBA3C*	NM_006001	NP_005992	440/451	LAALEKDYEEVGVDSVEAEAEEGEEY		
*TUBA3D*	NM_080386	NP_525125	440/451	LAALEKDYEEVGVDSVEAEAEEGEEY		
*TUBA3E*	NM_207312	NP_997195	435/451	LAALEKDCEEVGVDSVEAEAEEGEEY		
*TUBA4A*	NM_006000	NP_005991	432/450	MAALEKDYEEVGIDSYEDEDEGEE	*Tuba4a*	448/448
*TUBA8*	NM_018943	NP_061816	399/439	LAALEKDYEEVGTDSFEEENEGEEF	*Tuba8*	446/449
*TUBAL3*	NM_024803	NP_079079	329/444	LAALERDYEEVAQSF	*Tubal3*	369/446

Human genes identified by Khodiyar et al., 2007 are listed, along with accession IDs for DNA and protein. CTT sequences depict the last 15–27 genetically encoded amino acids. Underlined residue is the major site of polyglutamylation [63]. Gray denotes residues that differ from TUBA1A.

**Table 3:** Tuba1a mutations leading to lissencephaly spectrum phenotypes.

Reference	Mutation	Case Number	Gender/Age	Cortical Phenotype	Corpus Callosum Defect	Other Brain Malformations
[132]	**I5L**	not reported	F/7	perisylvian pachygyria	thin	brainstem mildly hypoplastic
[132]	**I5L**	not reported	F/2	perisylvian pachygyria	splenium hypoplastic	brainstem and cerebellum vermis mildly hypoplastic
[133]	**C25F**	K3373	M/2	lissencephaly, thin cortex	agenesis	poorly differentiated dysmorphic basal ganglia, slightly hypoplastic cerebellar vermis, ventricle dilation
[134]	E27Q *	not reported	F/0.5	simplified gyral pattern, diffuse pachygyria	hypoplastic	hypoplastic cerebellar vermis, lateral ventricle dilation
[15]	**E55K**	FR08-D5604	M/3	microcephaly, lissencephaly posterior, pachygyria anterior	partial agenesis	severe vermis hypoplasia, flattened isthmus and pons, hypoplastic hippocampus, trigones and occipital horns dilated
[20,21]	**T56M**	LIS_TUB_003_foetus18	M/24.3 GW	microcephaly, lissencephaly; absent cortical plate, 2 layered	complete agenesis	severe vermis hypoplasia; hypoplastic basal ganglia, severe hypoplasia of cerebellum and pons, optic nerve hypoplasia
[133]	R64W *	NCU_F41	F/3	extremely thin cerebral parenchyma	agenesis	optic nerve hypoplasia, hypoplastic brainstem, agenesis of cerebellum
[19]	**L70S**	not reported	F/2 weeks	lissencephaly, diffuse polymicrogyria-like	absent	cerebellar hypoplasia, enlarged lateral ventricles
[20]	**P72S**	LIS_TUB_012_foetus22	F/37.8 GW	severe lissencephaly	dysmorphic/hypoplastic	severe vermis hypoplasia
[16]	**L92V**	CM-66	fetus	lissencephaly with cerebellar hypoplasia; microcephaly	absent	small brainstem, cerebellum, and corticospinal tract, severe ventricular dilation
[20,21]	**N101S**	LIS_TUB_079_foetus25	M/25 GW	microcephaly, lissencephaly; 2–3 layered cortex, poorly differentiated	complete agenesis	severe vermis and hemispheric dysplasia; severe hypoplasia and dysplasia of cerebellum, severe hypoplasia of pons
[20]	**E113K**	LIS_TUB_031	M/11	central pachygyria	normal	normal cerebellum
[135]	R123C *	106115P	M	pachygyria	not reported	not reported
[16]	**V137D**	CM-107	not reported	pachygyria with cerebellar hypoplasia	absent	malformed hippocampus, thin brainstem, severe cerebellar hypoplasia
[135]	L152Q *	169451P	F	pachygyria	not reported	not reported
[12]	**I188L**	LIS_TUB_026	F/2	laminar heterotopia	thin, partial agenesis	vermis and brainstem hypoplasia, severe ventricular dilation
[136]	**C200Y**	not reported	F/8	lissencephaly	agenesis	abnormal hippocampus, dysmorphic and hypoplastic basal ganglia and thalamus, hypoplastic cerebellum, enlarged lateral ventricles
[132]	**Y210C**	not reported	M/1.5	lissencephaly anterior, pachygyria posterior	thin	brainstem and cerebellum vermis mildly hypoplastic
[137]	R214H	UW168-3	F/4	diffuse irregular gyration and sulcation	partial agenesis	hypoplasia of vermis, asymmetric pons, dysmorphic basal ganglia, cranial nerve hypoplasia, enlarged lateral ventricles
[16]	**D218Y**	LR07-213	not reported	lissencephaly with cerebellar hypoplasia, microcephaly	absent	thin brainstem, hypoplasia of cerebellar vermis
[137]	**I219V**	UW167-3	F/10	diffuse (L > R) irregular gyration and sulcation	partial agenesis	hypoplasia of vermis, asymmetric pons, dysmorphic basal ganglia, cranial nerve hypoplasia, enlarged lateral ventricles
[14]	**I238V**	LIS_TUB_022_foetus05	M/fetus	two-layered cortex, poorly differentiated	complete agenesis	disorganized hippocampus, internal capsule absent on one side, hypoplastic brainstem and corticospinal tract
[12]	**P263T**	not reported	M/fetus	lissencephaly, microcephaly	agenesis	abnormal hippocampus, cerebellum, vermis and brainstem hypoplasia, severe ventricular dilation
[14]	P263T	LIS_TUB_025_foetus06	M/fetus	two-layered cortex, poorly differentiated	complete agenesis	disorganized hippocampus, hypoplastic internal capsule, hypoplastic brainstem and corticospinal tract
[11]	**R264C**	not reported	not reported	pachygyria	agenesis	abnormal hippocampus, abnormal vermis, brainstem hypoplasia
[12]	R264C	LIS_TUB_037	M/4	pachygyria	present, abnormal shape	vermis and brainstem hypoplasia, mild ventricular dilation
[12]	R264C	LIS_TUB_036	M/2	pachygyria	present, abnormal shape	vermis hypoplasia, mild ventricular dilation
[13]	R264C	LIS_TUB_041	M/7	perisylvian pachygyria	posterior agenesis	severe dysgenesis of internal capsule
[13]	R264C	LIS_TUB_040	M/1.5	perisylvian pachygyria	mild hypoplasia	moderate dysgenesis of internal capsule
[20]	R264C	LIS_TUB_033	F/1.5	central pachygyria	normal	mild vermis hypoplasia
[20]	R264C	LIS_TUB_034	F/6.5	central pachygyria	hypogenetic	normal cerebellum
[20]	R264C	LIS_TUB_035	M/6	central pachygyria	normal	mild vermis hypoplasia
[138]	R264C	Patient B	M/2	grade 4 agyria, pachygyria (P > A gradient)	present, abnormal shape	hypoplastic basal ganglia
[20,21]	**R264H**	LIS_TUB_002_foetus20	F/24 GW	microcephaly, lissencephaly; absent cortical plate, 2 layered	complete agenesis	severe vermis hypoplasia; moderate hypoplasia of cerebellum, severe pons hypoplasia
[16]	**A270T**	LR07-244	not reported	pachygyria with cerebellar hypoplasia	absent	malformed hippocampus, thin brainstem, severe cerebellar hypoplasia
[139]	**A270S**	not reported	M/19 mo	mild posterior simplified cerebral gyral pattern	agenesis	severe hypoplastic cerebellar vermis, mildly dysplastic and hypoplastic cerebellar hemispheres, mildly hypoplastic brainstem, dysplastic basal ganglia, thalami, hypoplastic optic nerves, absent olfactory bulbs, lateral and third ventricle dilated
[12]	**L286F**	not reported	M/fetus	lissencephaly	agenesis	abnormal hippocampus, vermis and brainstem hypoplasia, severe ventricular dilation
[14]	L286F	LIS_TUB_007_foetus04	fetus	two-layered cortex, poorly differentiated	complete agenesis	absent hippocampus, olfactory bulb, and internal capsule, hypoplastic brainstem and corticospinal tract
[17]	**V303G**	LIS_TUB_006_foetus03	fetus	pachygyria, microcephaly	short and thin	thin brainstem, pons and medulla flattened, hypoplastic cerebellum and corticospinal tracts, severe ventricular dilation
[20,21]	**R320H**	LIS_TUB_005_foetus01	M/25 GW	microcephaly, lissencephaly; absent cortical plate	partial agenesis	severe vermis and hemispheric dysplasia/severe hypoplasia and dysplasia of cerebellum, severe hypoplasia (neuronal over migration) spinal cord anterior horn hypoplasia
[21]	R320H	LIS_TUB_081_foetus26	M/26 GW	absent cortical plate, 2 layered	complete agenesis	severe hypoplasia and dysplasia of cerebellum, severe hypoplasia of pons
[20,21]	**K326N**	LIS_TUB_004_foetus08	M/23 GW	microcephaly, lissencephaly; thick 2-layered cortex	partial agenesis; complete agenesis	severe vermis and hemispheric dysplasia/hypoplastic basal ganglia, severe hypoplasia and dysplasia of cerebellum, severe pons hypoplasia
[16]	**N329S**	LR05-388	not reported	lissencephaly with cerebellar hypoplasia, microcephaly	absent	thin brainstem, hypoplasia of cerebellar vermis
[138]	N329S	Patient A	M/0	grade 1 lissencephaly with cerebellar hypoplasia	agenesis	hypoplastic basal ganglia, cerebellum and brain stem
[20,21]	**V371E**	LIS_TUB_080_foetus24	F/23.3 GW	microcephaly, lissencephaly; 2–3 layered cortex, poorly differentiated	complete agenesis	severe vermis hypoplasia; hypoplastic basal ganglia, severe hypoplasia of cerebellum, severe pons hypoplasia
[140]	**A387V**	not reported	F/5	pachygyria with SBH	thin	simplified hippocampus, highly dysmorphic brainstem, flattened pons, mildly hypoplastic cerebellar vermis
[20]	**A369T**	LIS_TUB_030	M/11	central pachygyria	dysmorphic/hypoplastic	mild vermis hypoplasia
[13]	**L397P**	LIS_TUB_039	M/5.5	perisylvian pachygyria	posterior agenesis	severe vermis dysplasia, severe dysgenesis of internal capsule
[12]	**R402C**	not reported	M/fetus	lissencephaly	abnormally thick	abnormal hippocampus, vermis and brainstem hypoplasia, severe ventricular dilation
[15]	R402C	FR04-D4148	F/11	microcephaly, lissencephaly	thin, rostrum absent, splenium hypoplastic	mild vermis and pons hypoplasia, trigones and occipital horns dilated
[16]	R402C	LP95-073	not reported	lissencephaly	dysmorphic but intact	classic lissencephaly, round hippocampi with rounded rim
[16]	R402C	LR07-008	not reported	lissencephaly	dysmorphic but intact	classic lissencephaly, round hippocampi with rounded rim
[16]	R402C	LR06-210	not reported	lissencephaly	dysmorphic but intact	classic lissencephaly, round hippocampi with rounded rim
[16]	R402C	LR08-035	not reported	lissencephaly	dysmorphic but intact	classic lissencephaly, round hippocampi with rounded rim
[16]	R402C	LR06-064	not reported	lissencephaly	dysmorphic but intact	classic lissencephaly, round hippocampi with rounded rim
[20]	R402C	LIS_TUB_019	M/10	moderate lissencephaly	dysmorphic, hypoplastic	mild vermis hypoplasia
[14]	R402C	LIS_TUB_021_foetus07	M/fetus	thick four-layered cortex	abnormally thick and short	disorganized hippocampus, hypoplastic vermis, brainstem, abnormal corticospinal tract
[11,12]	**R402H**	LIS_TUB_023	M/11	lissencephaly	agenesis; thin, partial agenesis	abnormal vermis, brainstem hypoplasia/abnormal hippocampus, vermis and brainstem hypoplasia, severe ventricular dilation
[16]	R402H	LP97-039	not reported	lissencephaly with cerebellar hypoplasia	dysmorphic but intact	moderate cerebellar vermis hypoplasia, classic lissencephaly, round hippocampi with rounded rim,
[16]	R402H	LP97-041	not reported	lissencephaly with cerebellar hypoplasia	dysmorphic but intact	moderate cerebellar vermis hypoplasia, classic lissencephaly, round hippocampi with rounded rim
[20,21]	R402H	LIS_TUB_017_foetus02	M/29 GW	severe lissencephaly	complete agenesis	mild vermis and pons hypoplasia
[20]	R402H	LIS_TUB_014	M/4	severe lissencephaly	hypogenetic	severe hemispheric hypoplasia
[20]	R402H	LIS_TUB_015	M/1	severe lissencephaly	hypogenetic	severe hemispheric hypoplasia
[20]	R402H	LIS_TUB_016	F/1.5	severe lissencephaly	hypogenetic	vermis dysplasia
[141]	R402H	not reported	M/1	lissencephaly	not reported	severe dysplasia of brainstem and cerebellum
[15]	**R402L**	RE07-S1605	M/3	microcephaly, lissencephaly posterior, pachygyria anterior	thin	mild vermis and pons hypoplasia, retrocerebellar cyst, abnormal hippocampus, dilated lateral ventricles, occipital horns, anterior horns
[142]	R402L	not reported	F/1	microcephaly, lissencephaly posterior, pachygyria anterior	mild hypoplasia	severe hypoplasia of cerebellum, mild hypoplasia of brainstem, moderate ventricular dilation
[20,21]	**V409A**	LIS_TUB_011_foetus23	M/32 GW	severe lissencephaly with cerebellar hypoplasia	complete agenesis	severe vermis hypoplasia/severe hypoplasia of cerebellum and pons
[20]	**V409I**	LIS_TUB_032	M/10	central pachygyria	dysmorphic, hypoplastic	normal cerebellum
[12]	**S419L**	LIS_TUB_024	M/18	pachygyria	abnormal shape	abnormal hippocampus, vermis hypoplasia, severe ventricular dilation
[13]	**R422C**	LIS_TUB_042	F/4.5	perisylvian pachygyria	mild hypoplasia	mild vermis hypoplasia, severe dysgenesis of internal capsule
[13]	**R422H**	LIS_TUB_020	F/7	posterior pachygyria	mild hypoplasia	mild vermis hypoplasia, moderate dysgenesis of internal capsule
[15]	R422H	FR05-D4607	M/9	pachygyria with SBH	partial agenesis	severe vermis hypoplasia, dandy-walker malformation, hypoplasia of pons, abnormal hippocampus, dilated lateral ventricles, enlarged 4th ventricle
[15]	R422H	FR07-D5526	F/5	microcephaly, pachygyria with SBH	partial agenesis	moderate vermis hypoplasia, mild pons hypoplasia, abnormal hippocampus, dilated lateral ventricles, enlarged 4th ventricle
[16]	R422H	LR05-052	not reported	pachygyria with cerebellar hypoplasia	absent	malformed hippocampus, thin brainstem, severe cerebellar hypoplasia
[16]	R422H	LR08-340	not reported	pachygyria with cerebellar hypoplasia	absent	malformed hippocampus, thin brainstem, severe cerebellar hypoplasia
[20,21]	R422H	LIS_TUB_018_foetus10	F/28 GW	severe lissencephaly	complete agenesis	mild vermis and pons hypoplasia
[16]	**M425K**	LR08-388	not reported	lissencephaly with cerebellar hypoplasia, microcephaly	absent	thin brainstem, hypoplasia of cerebellar vermis
[20]	**E429Q**	LIS_TUB_001_foetus09	F/25 GW	microcephaly, lissencephaly	complete agenesis	severe vermis hypoplasia
[21]	E429Q	LIS_TUB_004_foetus09	F/25 GW	4 layered cortex	complete agenesis	hypoplastic basal ganglia, severe hypoplasia of cerebellum and pons
[13]	**G436R**	LIS_TUB_038	M/7	perisylvian pachygyria	mild hypoplasia	mild vermis hypoplasia, severe dysgenesis of internal capsule

**Bold** indicates first report of mutation; * Asterisk indicates mutation was identified through whole exome sequencing; Abbreviations: M, male; F, female; GW, gestational weeks SBH, subcortical band heterotopia.

**Table 4:** Tuba1a mutations leading to polymicrogyria phenotypes.

References	Mutation	Case Number	Gender/Age	Cortical Phenotype	Corpus Callosum Defect	Other Brain Malformations
[19]	**L70S**	not reported	F/2 weeks	lissencephaly, diffuse polymicrogyria-like	absent	cerebellar hypoplasia, enlarged lateral ventricles
[21]	P72S	LIS_TUB_012_foetus22	F/37.8 GW	unlayered generalized and asymmetric polymicrogyria	hypoplastic	severe hypoplasia of cerebellum and pons
[20]	**R123C**	LIS_TUB_044	F/3	central polymicrogyria-like cortical dysplasia	normal	vermis dysplasia
[20,21]	**S158L**	LIS_TUB_053_foetus21	F/24.5 GW	unlayered generalized and asymmetric polymicrogyria	complete agenesis	severe vermis and hemispheric dysplasia/hypoplastic basal ganglia, severe hypoplastic and dysmorphic cerebellum, hypoplasia olivary heterotopia
[18]	**Y161H**	LIS_TUB_047	F/11	asymmetrical perisylvian polymicrogyria	moderate hypoplasia	dysmorphic basal ganglia, dysplastic vermis and pons
[20,21]	**R214H**	LIS_TUB_043_foetus11	M/23 GW	central polymicrogyria-like cortical dysplasia; unlayered central and asymmetric polymicrogyria	complete agenesis	normal cerebellum; mild vermian hypoplasia, mild dysplastic olivary nuclei
[18]	**V235L**	LIS_TUB_046	M/7.5	asymmetrical perisylvian polymicrogyria	moderate hypoplasia	dysmorphic basal ganglia
[19]	**A333V**	not reported	M/7	right focal polymicrogyria-like	thin	vermis hypoplasia, mild brainstem hypoplasia, 4th ventricle enlarged
[20]	**V353I**	LIS_TUB_059	M/4	simplified gyral pattern with focal polymicrogyria	partial agenesis	normal
[18]	R390C	LIS_TUB_045	M/1	asymmetrical perisylvian polymicrogyria	severe hypoplasia	dysmorphic basal ganglia, dysplastic vermis, severe hypoplasia of brainstem
[147]	**R390H**	not reported	F/3	focal polymicrogyria	thin and incomplete	hypoplasia of left internal capsule, severe hypoplastic and asymmetric brainstem, vermis dysplasia, enlarged lateral ventricles
[20]	**D396Y**	LIS_TUB_078	F/4	central polymicrogyria-like cortical dysplasia	partial agenesis	severe vermis dysplasia

**Bold** indicates first report of mutation; Abbreviations: M, male; F, female; GW, gestational weeks.

**Table 5:** Tuba1a mutations leading to microcephaly phenotypes. Microcephaly classified as an OFC more than 2 SD below the appropriate mean (i.e., less than the 3rd percentile).

Reference	Mutation	Case Number	Gender/Age	Cortical Phenotype	OFC	Corpus Callosum Defect	Other Brain Malformations
[132]	**I5L**	not reported	F/7	perisylvian pachygyria	−2 SD	thin	brainstem mildly hypoplastic
[134]	E27Q *	not reported	F/5	simplified gyral pattern, diffuse pachygyria	−1 SD at birth, −3.3 SD at 2 mo.	hypoplastic	hypoplastic cerebellar vermis, lateral ventricle dilation
[15]	**E55K**	FR08-D5604	M/3	lissencephaly posterior, pachygyria anterior	−7 SD	partial agenesis	severe vermis hypoplasia, flattened isthmus and pons, hypoplastic hippocampus, trigones and occipital horns dilated
[20]	**T56M**	LIS_TUB_003_foetus18	M/24.3 GW	lissencephaly	microcephaly	complete agenesis	severe vermis hypoplasia
[21]	T56M	LIS_TUB_003_foetus18	M/24.3 GW	absent cortical plate, 2 layered	<3rd centile	complete agenesis	hypoplastic basal ganglia, severe hypoplasia of cerebellum and pons, optic nerve hypoplasia
[133]	R64W *	NCU_F41	F/3	extremely thin cerebral parenchyma	−2.4 SD	agenesis	optic nerve hypoplasia, hypoplastic brainstem, agenesis of cerebellum
[19]	**L70S**	not reported	F/2 weeks	lissencephaly, diffuse polymicrogyria-like	2–9 centile	absent	cerebellar hypoplasia, enlarged lateral ventricles
[21]	P72S	LIS_TUB_012_foetus22	F/37.8 GW	unlayered generalized and asymmetric polymicrogyria	5th centile	hypoplastic	severe hypoplasia of cerebellum and pons
[16]	**L92V**	CM-66	fetus	lissencephaly with cerebellar hypoplasia	microcephaly	absent	small brainstem, cerebellum, and corticospinal tract, severe ventricular dilation
[20]	**N101S**	LIS_TUB_079_foetus25	M/25 GW	lissencephaly	microcephaly	complete agenesis	severe vermis and hemispheric dysplasia
[21]	N101S	LIS_TUB_079_foetus25	M/25 GW	2–3 layered cortex, poorly differentiated	<3rd centile	complete agenesis	severe hypoplasia and dysplasia of cerebellum, severe hypoplasia of pons
[20]	**E113K**	LIS_TUB_031	M/11	central pachygyria	−3 SD	normal	normal cerebellum
[20]	**R123C**	LIS_TUB_044	F/3	central polymicrogyria-like cortical dysplasia	−3 SD	normal	vermis dysplasia
[18]	**Y161H**	LIS_TUB_047	F/11	asymmetrical perisylvian polymicrogyria	3rd centile	moderate hypoplasia	dysmorphic basal ganglia, dysplastic vermis and pons
[12]	**I188L**	LIS_TUB_026	F/2	laminar heterotopia	−4 SD	thin, partial agenesis	vermis and brainstem hypoplasia, severe ventricular dilation
[136]	C200Y * reported as C402Y	not reported	F/8	lissencephaly	−3.5 SD	agenesis	abnormal hippocampus, dysmorphic and hypoplastic basal ganglia and thalamus, hypoplastic cerebellum, enlarged lateral ventricles
[132]	**Y210C**	not reported	M/1.5	lissencephaly anterior, pachygyria posterior	−3 SD	thin	brainstem and cerebellum vermis mildly hypoplastic
[137]	R214H	UW168-3	F/4	diffuse irregular gyration and sulcation	<−2.5 SD	partial agenesis	hypoplasia of vermis, asymmetric pons, dysmorphic basal ganglia, cranial nerve hypoplasia, enlarged lateral ventricles
[16]	**D218Y**	LR07-213	Not reported	lissencephaly with cerebellar hypoplasia	microcephaly	absent	thin brainstem, hypoplasia of cerebellar vermis
[12]	**P263T**	not reported	M/fetus	lissencephaly	microcephaly	agenesis	abnormal hippocampus, cerebellum, vermis and brainstem hypoplasia, severe ventricular dilation
[12]	R264C	LIS_TUB_037	M/4	pachygyria	−4.5 SD	present, abnormal shape	vermis and brainstem hypoplasia, mild ventricular dilation
[12]	R264C	LIS_TUB_036	M/2	pachygyria	−4 SD	present, abnormal shape	vermis hypoplasia, mild ventricular dilation
[13]	R264C	LIS_TUB_041	M/7	perisylvian pachygyria	−4 SD	posterior agenesis	severe dysgenesis of internal capsule
[13]	R264C	LIS_TUB_040	M/1.5	perisylvian pachygyria	−4 SD	mild hypoplasia	moderate dysgenesis of internal capsule
[20]	R264C	LIS_TUB_033	F/1.5	central pachygyria	−5 SD	normal	mild vermis hypoplasia
[20]	R264C	LIS_TUB_034	F/6.5	central pachygyria	−4 SD	hypogenetic	normal cerebellum
[20]	R264C	LIS_TUB_035	M/6	central pachygyria	−3 SD	normal	mild vermis hypoplasia
[20]	**R264H**	LIS_TUB_002_foetus20	F/24 GW	lissencephaly	microcephaly	complete agenesis	severe vermis hypoplasia
[21]	R264H	LIS_TUB_002_foetus20	F/24 GW	absent cortical plate, 2 layered	<3rd centile	complete agenesis	moderate hypoplasia of cerebellum, severe pons hypoplasia
[139]	**A270S**	not reported	M/19 mo	mild posterior simplified cerebral gyral pattern	microcephaly	agenesis	severe hypoplastic cerebellar vermis, mildly dysplastic and hypoplastic cerebellar hemispheres, mildly hypoplastic brainstem, dysplastic basal ganglia, thalami, hypoplastic optic nerves, absent olfactory bulbs, lateral and third ventricle dilated
[14]	L286F	LIS_TUB_007_foetus04	fetus	two-layered cortex, poorly differentiated	<3rd centile	complete agenesis	absent hippocampus, olfactory bulb, and internal capsule, hypoplastic brainstem and corticospinal tract
[17]	**V303G**	LIS_TUB_006_foetus03	fetus	pachygyria	microcephaly	short and thin	thin brainstem, pons and medulla flattened, hypoplastic cerebellum and corticospinal tracts
[20]	**R320H**	LIS_TUB_005_foetus01	M/25 GW	lissencephaly	microcephaly	partial agenesis	severe vermis and hemispheric dysplasia
[21]	R320H	LIS_TUB_005_foetus01	M/25 GW	absent cortical plate	<3rd centile	partial agenesis	severe hypoplasia and dysplasia of cerebellum, severe hypoplasia (neuronal over-migration) spinal cord anterior horn hypoplasia
[21]	R320H	LIS_TUB_081_foetus26	M/26 GW	absent cortical plate, 2 layered	<3rd centile	complete agenesis	severe hypoplasia and dysplasia of cerebellum, severe hypoplasia of pons
[20]	**K326N**	LIS_TUB_004_foetus08	M/23 GW	lissencephaly	microcephaly	partial agenesis	severe vermis and hemispheric dysplasia
[21]	K326N	LIS_TUB_004_foetus08	M/23 GW	thick 2-layered cortex	<3rd centile	complete agenesis	hypoplastic basal ganglia, severe hypoplasia and dysplasia of cerebellum, severe pons hypoplasia
[16]	**N329S**	LR05-388	Not reported	lissencephaly with cerebellar hypoplasia	microcephaly	absent	thin brainstem, hypoplasia of cerebellar vermis
[20]	**V353I**	LIS_TUB_059	M/4	simplified gyral pattern with focal polymicrogyria	−2 SD	partial agenesis	normal
[20]	**V371E**	LIS_TUB_080_foetus24	F/23.3 GW	lissencephaly	<3rd centile	complete agenesis	severe vermis hypoplasia
[21]	V371E	LIS_TUB_080_foetus24	F/23.3 GW	2–3 layered cortex, poorly differentiated	<3rd centile	complete agenesis	hypoplastic basal ganglia, severe hypoplasia of cerebellum, severe pons hypoplasia
[140]	**A387V**	Not reported	F/5	pachygyria with SBH (subcortical band heterotopia)	<3rd centile	thin	simplified hippocampus, highly dysmorphic brainstem, flattened pons, mildly hypoplastic cerebellar vermis
[18]	R390C	LIS_TUB_045	M/1	asymmetrical perisylvian polymicrogyria	microcephaly	severe hypoplasia	dysmorphic basal ganglia, dysplastic vermis, severe hypoplasia of brainstem
[20]	**A369T**	LIS_TUB_030	M/11	central pachygyria	−2 SD	dysmorphic, hypoplastic	mild vermis hypoplasia
[20]	**D396Y**	LIS_TUB_078	F/4	central polymicrogyria-like cortical dysplasia	−3 SD	partial agenesis	severe vermis dysplasia
[13]	**L397P**	LIS_TUB_039	M/5.5	perisylvian pachygyria	−4 SD	posterior agenesis	severe vermis dysplasia, severe dysgenesis of internal capsule
[15]	R402C	FR04-D4148	F/11	lissencephaly	−4 SD	thin, rostrum absent, splenium hypoplastic	mild vermis and pons hypoplasia, trigones and occipital horns dilated
[20]	R402C	LIS_TUB_019	M/10	moderate lissencephaly	−3 SD	dysmorphic, hypoplastic	mild vermis hypoplasia
[12]	R402H	LIS_TUB_023	M/11	lissencephaly	−3 SD	thin, partial agenesis	abnormal hippocampus, vermis and brainstem hypoplasia, severe ventricular dilation
[20]	R402H	LIS_TUB_014	M/4	severe lissencephaly	−4 SD	hypogenetic	severe hemispheric hypoplasia
[20]	R402H	LIS_TUB_015	M/1	severe lissencephaly	−4 SD	hypogenetic	severe hemispheric hypoplasia
[20]	R402H	LIS_TUB_016	F/1.5	severe lissencephaly	−3 SD	hypogenetic	vermis dysplasia
[141]	R402H	not reported	M/1	lissencephaly	microcephaly		severe dysplasia of brainstem and cerebellum
[15]	**R402L**	RE07-S1605	M/3	lissencephaly posterior, pachygyria anterior	−3.5 SD	thin	mild vermis and pons hypoplasia, retrocerebellar cyst, abnormal hippocampus, dilated lateral ventricles, occipital horns, anterior horns
[142]	R402L	Not reported	F/1	lissencephaly posterior, pachygyria anterior	−4 SD	mild hypoplasia	severe hypoplasia of cerebellum, mild hypoplasia of brainstem
[21]	V409A	LIS_TUB_011_foetus23	M/32 GW	not available	5th centile	complete agenesis	severe hypoplasia of cerebellum and pons
[20]	**V409I**	LIS_TUB_032	M/10	central pachygyria	−2 SD	dysmorphic, hypoplastic	normal cerebellum
[13]	**R422C**	LIS_TUB_042	F/4.5	perisylvian pachygyria	−3 SD	mild hypoplasia	mild vermis hypoplasia, severe dysgenesis of internal capsule
[13]	**R422H**	LIS_TUB_020	F/7	posterior pachygyria	−4 SD	mild hypoplasia	mild vermis hypoplasia, moderate dysgenesis of internal capsule
[15]	R422H	FR05-D4607	M/9	pachygyria with SBH	−4 SD	partial agenesis	severe vermis hypoplasia, dandy-walker malformation, hypoplasia of pons, abnormal hippocampus
[15]	R422H	FR07-D5526	F/.5	pachygyria with SBH	−3.5 SD	partial agenesis	moderate vermis hypoplasia, mild pons hypoplasia, abnormal hippocampus
[16]	**M425K**	LR08-388	Not reported	lissencephaly with cerebellar hypoplasia	microcephaly	absent	thin brainstem, hypoplasia of cerebellar vermis
[20]	**E429Q**	LIS_TUB_001_foetus09	F/25 GW	lissencephaly	microcephaly	complete agenesis	severe vermis hypoplasia
[21]	E429Q	LIS_TUB_004_foetus09	F/25 GW	4 layered cortex	<3rd centile	complete agenesis	hypoplastic basal ganglia, severe hypoplasia of cerebellum and pons
[13]	**G436R**	LIS_TUB_038	M/7	perisylvian pachygyria	−3 SD	mild hypoplasia	mild vermis hypoplasia, severe dysgenesis of internal capsule

**Bold** indicates first report of mutation; * Asterisk indicates mutation was identified through whole exome sequencing; Abbreviations: OFC, occipitofrontal circumference; M, male; F, female; GW, gestational weeks; SD, standard deviation; SBH, subcortical band heterotopia.

**Table 6:** Studies of *TUBA1A* expression during mouse development.

			Developmental Time Point							
			E9.5	E10.5	E11.5	E12	E12.5	E13.5	E14.5	E16.5
**Developing CNS**	brain	brain, unspecified	[159]		[156]			[156]		
		forebrain	[158]							
		midbrain	[158]							
		hindbrain	[158]							
		telencephalon								[12]
		diencephalon		[156]	[156]			[156]		[12]
		mesencephalon								[12]
		metencephalon		[156]	[156]			[156]		[12]
		myelencephalon		[156]	[156]			[156]		[12]
		developing neocortex					[158]	[156]	[156]	[12]
		developing striatum					[159]			
		developing hippocampus					[159]			
		developing thalamus					[159]			
		developing amygdala					[159]			
		developing hypothalamus					[159]			
		developing cerebellum					[159]			[12]
		developing brain stem								[12]
		spinal cord	[158]	[156]	[156]		[159]	[156]		
		retina				[158]		[156]		
**Developing PNS**	cranial nerves	cranial ganglia			[156]			[156]		
		trigeminal ganglion	[158]							
		fascioacoustic ganglion	[158]							
		glossopharyngeal ganglia		[158]						
		developing olfactory bulbs			[156]		[159]	[156]		
		nasal epithelium		[158]						
		vomeronasal organ			[158]					
		dorsal root ganglia		[158]	[156]			[156]		[12]
		sensory ganglia			[156]			[156]		
	ANS	sympathetic ganglia		[158]	[156]			[156]		
		parasympathetic ganglia			[156]			[156]		
		heart	[158]							

**Table 7:** Studies of *TUBA1A* expression in postnatal mouse.

			Postnatal Time Point							
			P0	P3	P6	P10	P15	P22	P32	Adult
**CNS**	brain	brain, unspecified		[10]	[10]	[10]	[10]	[10]	[10]	[10,157]
		olfactory system	[159]							[157,159]
		cortex	[159]			[12]				[157]
		corpus callosum	[159]							[159]
		striatum	[159]							
		rostral migratory stream	[159]			[12]				
		basal ganglia								[157]
		basal forebrain								[157]
		hippocampus	[159]			[12]				[157,159]
		amygdala	[159]							[157,159]
		hypothalamus	[159]							[157,159]
		thalamus	[159]							[157,159]
		subthalamus								[157]
		midbrain	[159]							[157,159]
		pons								[157]
		medulla								[157]
		cerebellum	[159]			[12]				[157]
		brainstem				[12]				[157]
		spinal cord	[159]							[159]
**Other**		lung		[10]	[10]	[10]	[10]	[10]	[10]	[10]
		testes				[10]			[10]	[10]
